# Modular Reorganization of Signaling Networks during the Development of Colon Adenoma and Carcinoma

**DOI:** 10.1021/acs.jpcb.0c09307

**Published:** 2021-02-09

**Authors:** Klára Schulc, Zsolt T. Nagy, Sebestyén Kamp, János Molnár, Daniel V. Veres, Peter Csermely, Borbála M. Kovács

**Affiliations:** † Department of Molecular Biology, Semmelweis University, Budapest 1085, Hungary; ‡ Turbine Ltd, Budapest, Hungary

## Abstract

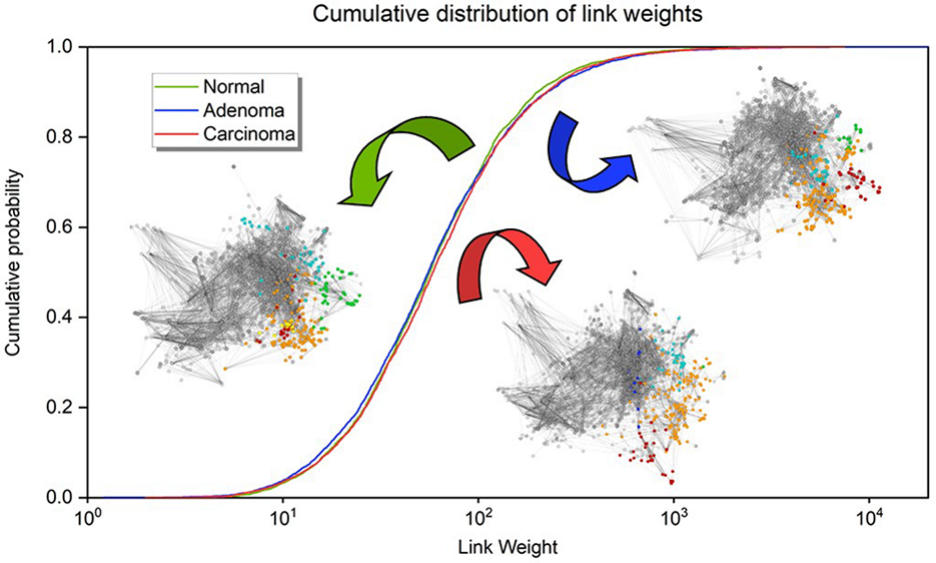

Network science is an emerging tool in systems biology and oncology, providing novel, system-level insight into the development of cancer. The aim of this project was to study the signaling networks in the process of oncogenesis to explore the adaptive mechanisms taking part in the cancerous transformation of healthy cells. For this purpose, colon cancer proved to be an excellent candidate as the preliminary phase, and adenoma has a long evolution time. In our work, transcriptomic data have been collected from normal colon, colon adenoma, and colon cancer samples to calculating link (i.e., network edge) weights as approximative proxies for protein abundances, and link weights were included in the Human Cancer Signaling Network. Here we show that the adenoma phase clearly differs from the normal and cancer states in terms of a more scattered link weight distribution and enlarged network diameter. Modular analysis shows the rearrangement of the apoptosis- and the cell-cycle-related modules, whose pathway enrichment analysis supports the relevance of targeted therapy. Our work enriches the system-wide assessment of cancer development, showing specific changes for the adenoma state.

## 1 Introduction

Colon cancer is a malignant tumor originating in the large intestine, which is histologically considered to be an adenocarcinoma in 95% of the cases. The relevance of this disease is hard to underestimate, as colorectal cancer (CRC) is the third most common cancer diagnosed in males and the second in females. Although the survival rate is increasing in the United States, the mortality of the disease is still among the leading cancer subtypes according to the GLOBOCAN database of the World Health Organization.^1^ Like most civilizational diseases, the etiology of colon cancer is multifactorial, involving both genetic (e.g., familial adenomatous polyposis, Lynch Syndrome) and environmental (lifestyle risk factors such as alcohol and smoking) causes. Benign polyps/adenomas are extremely common, and regretfully, 10% of them transform malignantly. Nearly all CRC develop this way, thus an understanding of the process is essential. There are heterogeneous causes of colon cancer development occurring at the molecular level, involving epigenomic and genomic instabilities, resulting in the deregulations of various signaling pathways involved in cell differentiation and growth.^2^ Despite thousands of mutant genes, 15 driver mutations were identified as key features in the pathogenesis of colon cancer.^3^ The treatment of the disease mainly includes surgery and chemotherapy,^4^ but targeted therapies such as EGFR and VEGFR inhibition and immunotherapy came into view as palliative treatment options.^5^

In recent years, the role of network science in medicine and molecular biology has been growing extensively.^6−9^ As technological development allowed the detailed measurement of mRNA expression, networks were constructed to perform data analysis to provide information about signaling in normal and pathological stages of a cell on the system level. Detailed network topology studies have been conducted in which the network diameter was calculated and modular structure was detected via different algorithms.^10−23^ In our earlier joint studies with the pioneering expert in the field, Prof. Ruth Nussinov^9,24−26^ showed that signaling networks proved to be very valuable tools in characterizing the development of cancer and in the discovery of new therapeutic modalities. Signaling networks are able to describe the complex crossroads of signal transduction pathways in a clearly understandable way. For this reason, the Human Cancer Signaling Network,^27^ which is a signaling network enriched by relating protein–protein interactions, appeared to be an excellent candidate for studying colon cancer, one of the most common oncological diseases in the world.^1^

Network topological analysis focusing on highly overlapping modules has never been applied before to colon cancer data. The modularization approach we used focuses on extensively overlapping and hierarchical modules and provides a powerful way to model cellular processes such as the functional reorganization of protein complexes.^28^ This sophisticated separation of modules was required for the results to show the deregulation of the apoptotic process and the increasing activity of the cell cycle.

This article demonstrates that the adenoma network forms a distinguished state between the normal and carcinoma networks. This statement is supported by the findings that the adenoma network has the highest standard deviation in the distribution of the link weights and has the largest diameter. Regarding the changes in the biological functions, the reorganized modules are in the areas of apoptosis regulation and the cell cycle, which are both well-known features of carcinogenesis. In terms of clinical relevance, the Gene Ontology^29,30^ pathway enrichment analysis resulted in confirming the strengthening role of the EGFR and VEGFR, whose inhibitors are already in use as palliative treatment options for colorectal cancer. The significant achievement of the work presented here is that it shows that using a highly overlapping modularization method enables us to model the construction of biological systems, opening new possibilities to understanding the pathogenesis of cancer.

## 2 Materials and Methods

### 2.1 Concept of the Analysis

Building and analyzing networks from the significant fold changes between the expression levels of two biological conditions is a common method. However, with the help of the mRNA abundance values, each three stages of carcinogenesis (normal, adenoma, and carcinoma) can be modeled individually. Moreover, with the use of a standard, previously described network model (i.e., The Human Cancer Signaling Network,^27^ see Materials and Methods) a complex picture can be used to represent the biological states so that they become analyzable on their own, not just their relationship with each other. With the inclusion of nonsignificantly changing data in the analysis, fewer apparent global and mesoscopic changes can be identified among the three networks. This work focuses on the structural reorganization of the network modules, for which it is inevitable to understand their biological function. In our model, modules mainly represent protein complexes. The biological role of a protein complex is determined by all members; therefore, the structural reorganization in itself, without any significant fold change, can be informative, as shown in this article.

### 2.2 Human Cancer Signaling Network

The network used for the colon cancer gene expression analysis was the Human Cancer Signaling Network constructed by Cui et al.^27^ It was built after a comprehensive analysis of cancer signaling from genes that were frequently mutated in cancer based on, inter alia, the COSMIC,^31^ BioCarta,^32^ and Cancer Cell Map databases. This network contains 1634 nodes (that mainly represent proteins) and 5089 directed links that include 2403 activating links, 741 inhibiting links, 1915 undirected (association-type or interaction-based) links, and 30 links of unknown types. After mapping this network with the gene expression data set and deleting the interactions that were not covered by it or whose link type was not known and the nodes that were not connected to the giant component of the network, 1600 nodes and 5060 links remained, a network large enough not only for the analysis of local alterations but also for that of global topological changes during carcinogenesis.

### 2.3 Mapped Gene Expression Omnibus Data Sets

The healthy, adenomatous, and carcinomatous colon transcriptomic data for this study were extracted from the Gene Expression Omnibus database. The GSE20916,^33^ GSE4183,^34^ GSE8671,^35^ GSE37364,^36^ and GSE33113^37^ series were processed, with the gene expression data of altogether 437 samples from patients of various ethnicities. Out of these samples, 128 were classified as healthy colons, 131 as adenomas, and 178 as colon adenocarcinomas. (See Table S1.) Data were collected with differentiation between left- and right-sided colon cancer. To avoid internal heterogeneity, the data sets selected contain mainly left-sided samples, which are known to be less immunogenic and richer in cancer-signaling-related mutations. The clinical stage in the colon cancer samples were diversely collected, aiming for a “general cancer phenotype”.

### 2.4 Conversion of mRNA Expression Data to Link Weights

After collecting the data, RNA normalization and median aggregation were conducted for each of the mRNA data points. The median of every GEO^29,30^ gene expression data set (which we refer to as abundance) in all three states (normal, adenoma, and carcinoma) was calculated respectively for each of the genes. While mapping the data to the structure of the Human Cancer Signaling Network,^27^ the link weights were calculated by multiplying the abundances of the two nodes belonging to a given link:
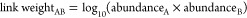
The reason for this formula lies in the assumption that multiplication can highlight the upregulated genes in cancer. Additionally, because a significant part of the Human Cancer Signaling Network^27^ is based on protein–protein interactions, in this case the probability of the attachment correlates with the multiplied abundances of the two interacting proteins.^38−40^ For further calculations, the link weights were logarithmically transformed in order to avoid the distortions caused by some highly outlying gene expression values. The result was three sets of link weights representing the three data sets collected from normal colon, colon adenoma, and colon adenocarcinoma samples. The abstract models of these biological entities were defined in the text as a normal network for normal, healthy colons, an adenoma network for adenomatous colons, and a carcinoma network for cancerous colons.

In this study, mRNA measures were used as a proxy for protein abundance. The authors are aware that this is only an approximation, but the current lack of ample high-quality and extensive proteomic data justifies this estimation. Link weights (calculated as the product of node abundances) therefore characterize the probability of the given signaling interaction, which is high when the abundance of interacting nodes is high and low when it is low.

While microarray data are known to be quite noisy,^33^ signaling networks are known to be quite robust to noise.^9^ To show the robustness of the results, all significant measurements were performed with artificially added noise by randomly increasing or decreasing the abundances by 5%. (For further details, see Text S1.)

### 2.5 Calculation of Network Diameter

For the three different networks, three different cases were considered: undirected, directed, and mixed networks. Undirected and directed cases contained only undirected and directed links, respectively. The mixed network contained both directed and undirected links, and the undirected links were substituted with two directed links of the same link weight. The calculation of the network diameter was conducted via the NetworkX Python package,^41^ which is widely used for network analysis. Because NetworkX cannot calculate the network diameter of a non-singly connected component, removing the isolated parts was the next step in processing. Since NetworkX considers link weights to be path lengths between two nodes, transformation of the link weights was necessary for the appropriate representation of the probability of the biological connection between the nodes in the diameter calculation. As a next step, negative logarithmic mapping was performed so that the shortest distance mapped into the most likely transition path. The computation of the diameter was then executed with the help of the built-in Dijkstra algorithm^42^ of NetworkX. The last step was choosing the longest of the calculated shortest paths and summing the link weights belonging to each of the links. Considering the fact that although the network diameter is traditionally defined as the longest of the shortest paths, in the case of weighted networks the average path length may be more reliable, which was also calculated. In summary, the treatment of the undirected link weights in the mixed and undirected networks may be considered to be an improvement in the diameter calculation. For further details, see Codes S1–S3.

### 2.6 EntOpt Layout Cytoscape Plugin

The EntOpt Layout program^43^ is a network visualization plugin of the Cytoscape software environment for the analysis of biomolecular interaction networks. Its principle is relative entropy optimization.^44^ In other words, it aims to arrange networks in a way that involves the least information loss (i.e., relative entropy), resulting in an easily interpretable and visually pleasing appearance. Entropy-based visualization also helps to highlight the global differences between the specific states in the networks, as the change in the link weights and the strengthening or weakening of different regions affect the visual output of the program. The EntOpt Layout is a unique tool for visualizing modules by grouping nodes together with a locally higher link ratio, also for weighted networks. Our aim was to visualize the pre-established modules (detected with the ModuLand plugin, see Section 2.7), where the nodes usually have similar biological functions. Therefore, with the use of the square adjacency matrix function, the nodes were put together on the basis of the neighborhood similarity, so nodes close to each other are not necessarily connected. We used the recommended visualization settings provided by the authors of the EntOpt Layout to display the Human Cancer Signaling Network^27^ which are detailed in Text S2 and ref (43).

### 2.7 ModuLand Cytoscape Plugin

The ModuLand plugin^45^ of Cytoscape is a Java-based network analyzing program focused on extensively overlapping modules and hierarchical layers (Figures S1–S3). This embeddedness and overlap in terms of the division of labor between functional units such as protein complexes is widely spread in living, real-world networks (e.g., cells). In this work, the authors used biological networks to model protein–protein interactions and signaling cascades of cells. For this reason, the ModuLand plugin was a useful tool for understanding the functional modular changes comparing the three networks (normal, adenoma, and carcinoma networks). It calculates the assignment strength of each node to each discovered module based on influence functions and centrality, creating a community landscape of overlapping hills (modules) of nodes. One of the main outputs of the plugin is the community centrality measure, which determines the sum of local influence zones for each node, also representing the whole network’s influence on one of its nodes^28^ Nodes having the largest community centrality measure in the module they mostly belong to are the main integrators and organizers of their module. On the basis of this property, estimating the biological function of modules by their leading nodes in their community centrality measure is a more precise way than checking each node in the module separately. The program also calculates several other topological measures, such as the modular overlap and effective degree.

## 3 Results

### 3.1 Link Weight Changes

To better understand the global topological changes in the network during carcinogenesis, the differences in the link weight data (calculated as a product of node abundances, see Materials and Methods) were analyzed in the three states of the network. To emphasize the importance of the differences among the three data sets, the authors note that the structure of the network remains the same in normal, adenoma, and carcinoma states, and the diversity of the results stems only from the change in the mRNA expression data. For easier comprehension, the authors considered the normal network to be a model network of a normal, healthy colon, the adenoma network to be that of a colon adenoma, and the carcinoma network to be that of a model network of colon adenocarcinoma.

**Figure 1:**
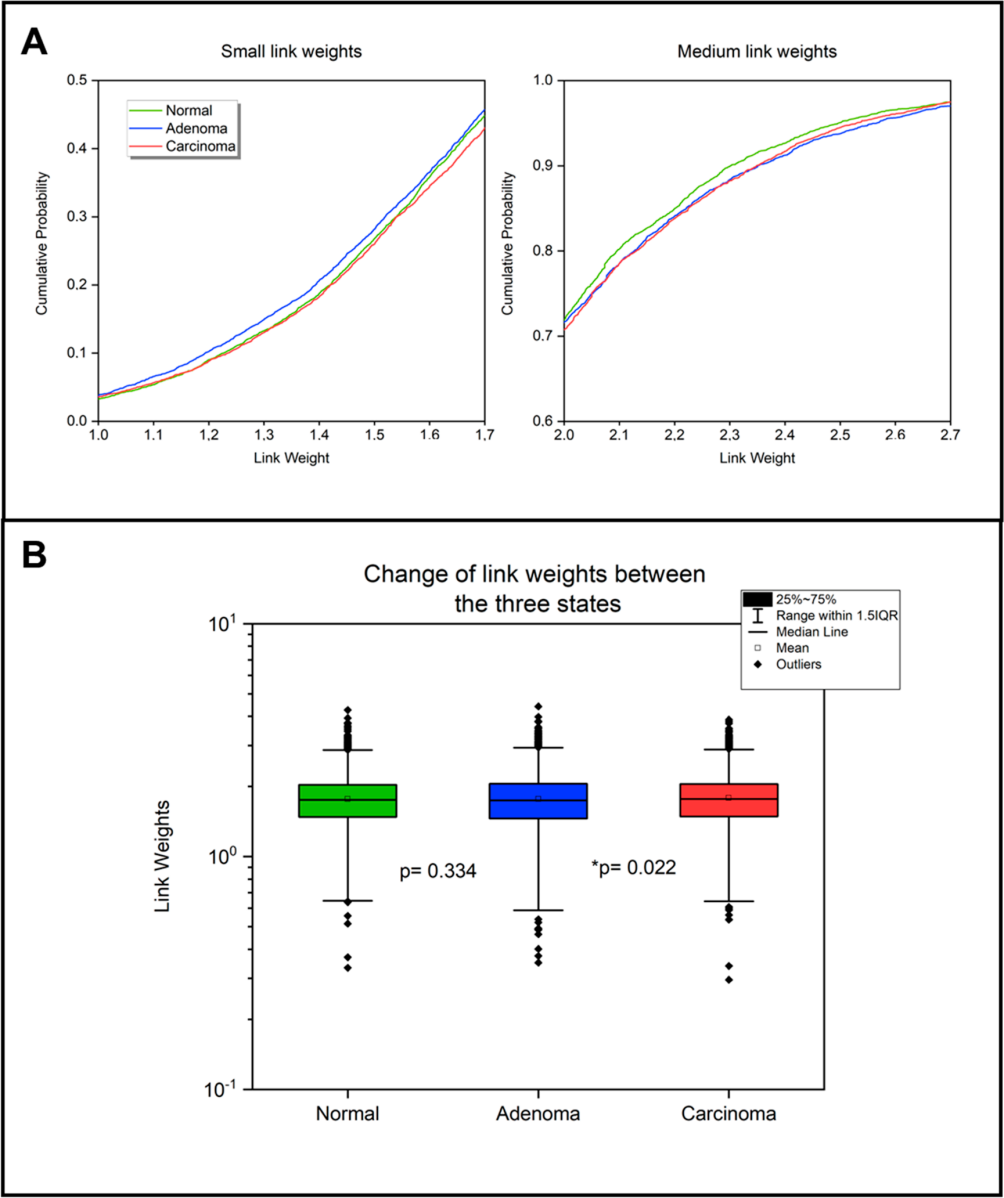
Link weight changes between the normal colon, colon adenoma, and colon carcinoma networks. The networks and link weights were created as described in the Materials and Methods section. (A) Cumulative probability distribution of the link weights in the three different states. In the two graphs, link weights from 1 to 1.7 and from 2 to 2.7 are highlighted. The first graph shows that link weights of the adenoma network have a higher cumulative probability, meaning that the number of smaller link weights (defined as a link weight of less than 2) is higher than in normal and carcinoma networks. On the second graph, the normal network’s cumulative probability values exceed those of the others, which indicates that in the normal network the medium-weighted links (defined as a link weight of between 2 and 2.7) are more accentuated. (B) Box plots of the link weight data of the three different states. The *p* values between each two networks (normal–adenoma and adenoma–carcinoma; paired Wilcoxon test) are highlighted on the panel. These results together (along with the changes regarding the mean and median values; see the main text) suggest that the weakest links are becoming stronger in the carcinoma network, thus showing the realignment of the network in cancer.

The distribution of the link weights (Figure 1A and Figure S4) shows a slightly different pattern in the three networks representing each biological state, which is further demonstrable with data binning (Figures S5 and S6). Among the weak links (defined here as link weights of less than 2), the adenoma network has the highest cumulative probability, demonstrating lower link weights (minimum is 0.0797) compared to the other states. Normal and carcinoma networks both have larger minimum link weights (0.333 and 0.296, respectively), indicating a similarity in normal and carcinoma networks. The normal network has the highest cumulative probability among the medium-strength links (link weights of between 2 and 2.7), thus having the lowest third quartile (2.035) and interquartile range (0.556). Strong links (link weights greater than 2.7) are very prominent in the adenoma network (maximum link weight of 4.414) and less expressed in the carcinoma network (maximum link weight of 3.87), while the normal network is in between the carcinoma and adenoma networks. Nonlogarithmic link weights and abundances also show these differences, while they are less accentuated with the weighted degree distribution (Figures S7–S9). These results are also robust to noise (Figures S10 and S11).

The differences that are found can also be characterized by the changes in the median, which is the smallest in the adenoma network (1.743), the largest in the carcinoma network (1.767), and in between in the normal network (1.7505), as visible in the box plot of the link weights of the three networks (Figure 1B). The box plot also demonstrates the standard deviations: in the adenoma network, it is the largest (0.4602) and much smaller in the normal and carcinoma networks (0.43349 and 0.44459, respectively). The differences among the link weight distributions of the three networks are also significant (paired Wilcoxon test; *p* < 0.0001), even with 5% added noise (paired Wilcoxon test; *p* < 0.0001). These small but consequent global differences highlight subtle changes among the three networks. In the next sections, with the use of more link-weight-sensitive methods, the functional implications of these changes have been explored. In conclusion, normal and carcinoma networks seem to have a very similar link weight distribution among the small and large link weights, but among the medium link weights, the normal network becomes the most prominent.

### 3.2 Network Diameter

The network diameter by definition is the sum of the link weights within the longest shortest path between two nodes.^46^ However, in order to calculate this, a nonconventional approach was necessary. In the processed molecular networks, weights are considered to be the likelihood of the interaction between two nodes (which represent molecules in this case); therefore, the higher the weight value, the shorter the path, while in graph theory, weights are considered to be lengths. To overcome this issue, during the calculation of the longest shortest path, the link weights were negated, and after running the Dijkstra algorithm,^42^ the weighted network diameter was calculated by summing the negatively mapped link weights in the longest shortest path of the network (further details in Materials and Methods). For further evaluation of the results, the average shortest path length was calculated with the same method.

Link directivity raised another complex methodical question concerning whether the direction of the connections should be included in the calculation. Ultimately, three methods were used. First, the network was considered to be completely undirected, and then the directivity of activating and inhibiting links was respected but not that of undirected links. Finally, a mixed graph was created by duplicating undirected links by taking them as two different connections pointing in the opposite direction (Table 1).

**Table 1:** Network Diameters in the Normal, Adenoma, and Carcinoma Networks with Negative Logarithmic Mapping

	undirected^a^		directed^b^		mixed graph^c^	
	network diameter^d^	average path length^e^	network diameter^d^	average path length^e^	network diameter^d^	average path length^e^
normal	34.361	9.984	36.988	12.940	36.435	11.397
adenoma	36.365	10.667	40.051	13.790	39.152	12.198
carcinoma	29.753	8.224	32.499	10.798	30.769	9.423

a In the network, every link was assigned as undirected.

b The original directivity was preserved in the calculation.

c Mixed graphs were defined as directed graphs, where undirected links were considered to be bidirectional links.

d Network diameters were calculated with the Dijkstra algorithm.

e Average path lengths were calculated with the NetworkX package.

Out of all three networks, the adenoma network has the largest and the carcinoma network has the smallest network diameter by all calculation methods. The average shortest path length changes accordingly. The same results were received with two different approaches and also with additional noise (Table S2). The enlarged network diameter in the adenoma network may imply a less compact network structure. The shrinkage of the network diameter and average shortest path length in the carcinoma network may be an adaptation strategy by making the information flow in the network more effective, which is already observed in other topological studies. This result may represent the cellular response to stress in a cancerous environment, offering new possibilities for interpretation compared to changes during carcinogenesis.

### 3.3 Functional Changes in the Modules

With the discrete module assignment method, the ModuLand plugin assigns the nodes to the module to which they mostly belong.^45^ The name of the module often depicts its function, but for a more precise estimation of the biological functions, the nodes with the highest community centrality measure within the module were analyzed. Community centrality refers to the local influence zone of each node and thus helps in the determination of a given node’s dominance within its neighborhood.^28^ The nodes chosen with the above-described approach were checked in the UniProt database^47^ (in which each node refers to a protein in the network), and the summarized biological functions were set to be the functions of the modules.

**Figure 2:**
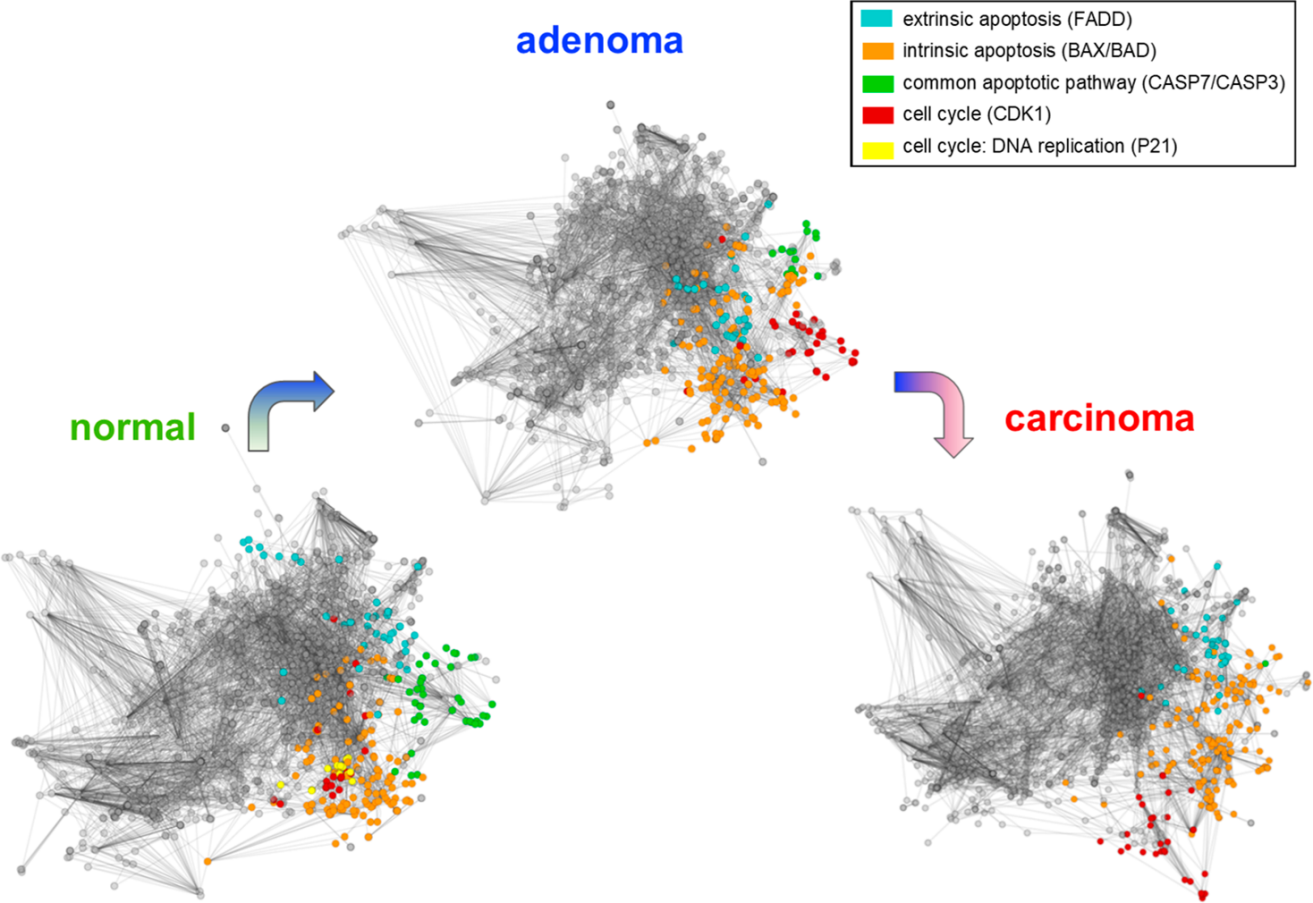
Functional changes in the modules of apoptosis and the cell cycle. The Human Cancer Signaling network was visualized using the EntOpt Cytoscape plugin.^40^ The most relevant modular changes were chosen and colored. The functions of the modules were estimated from the community centrality measure of the ModuLand plugin^45^ (Materials and Methods). The module responsible for the common apoptotic pathway (named CASP3 in normal and CASP7 in adenoma networks) is continuously melting into the intrinsic apoptotic pathway module (named BAD in the normal network and BAX in adenoma and carcinoma networks), while the structure and size of the extrinsic apoptotic module remain stable (named FADD). The module responsible for controlling the cell cycle and DNA replication (named P21) melts into the CDK1 module. A small part remains separate, named the PCNA module. (For details, see Table S3.) These functional changes suggest that the control of the apoptotic process and the cell cycle becomes less organized and a less important part of the network, in line with our knowledge of tumor biology. These results showed a robustness to noise (Table S4).

The most important modular changes in the network were in modules associated with cell cycle and apoptosis (Figure 2). The appropriate regulation of these functions is essential to the survival of cancer cells. One of the most important network changes in the process of carcinogenesis is that the module responsible for the final common pathway of apoptosis (called CASP3 in the normal network and CASP7 in the adenoma network) gradually merges with the module responsible for intrinsic apoptosis (called BAX), suggesting a decrease in the importance of apoptotic functions. The p21 protein is responsible for cell cycle arrest by inhibiting cyclin-dependent kinases, also acting as an effector in the DNA damage-induced p53-mediated apoptosis in colon adenocarcinoma.^48^ Thus, the fusion of the module organized around p21 in the CDK1 module in the adenoma and carcinoma networks indicates the loss of function of cell cycle control during these pathologic transitions.

Interestingly, the EntOpt images of normal and carcinoma networks are very similar to each other, and the adenoma network displays a slightly more compact network periphery compared to the other two networks (Figures S12–S15). This observation may correlate with the findings that several topological parameters of the carcinoma network are closer to those of the normal network than to the topological parameters of the adenoma network.

There was a further investigation into the change in the modular overlap among the three networks, but this analysis did not bring about significant differences. (For further details, see Text S3 and Figures S16–S19.)

### 3.4 Strongest and Weakest Links in the Network

**Figure 3:**
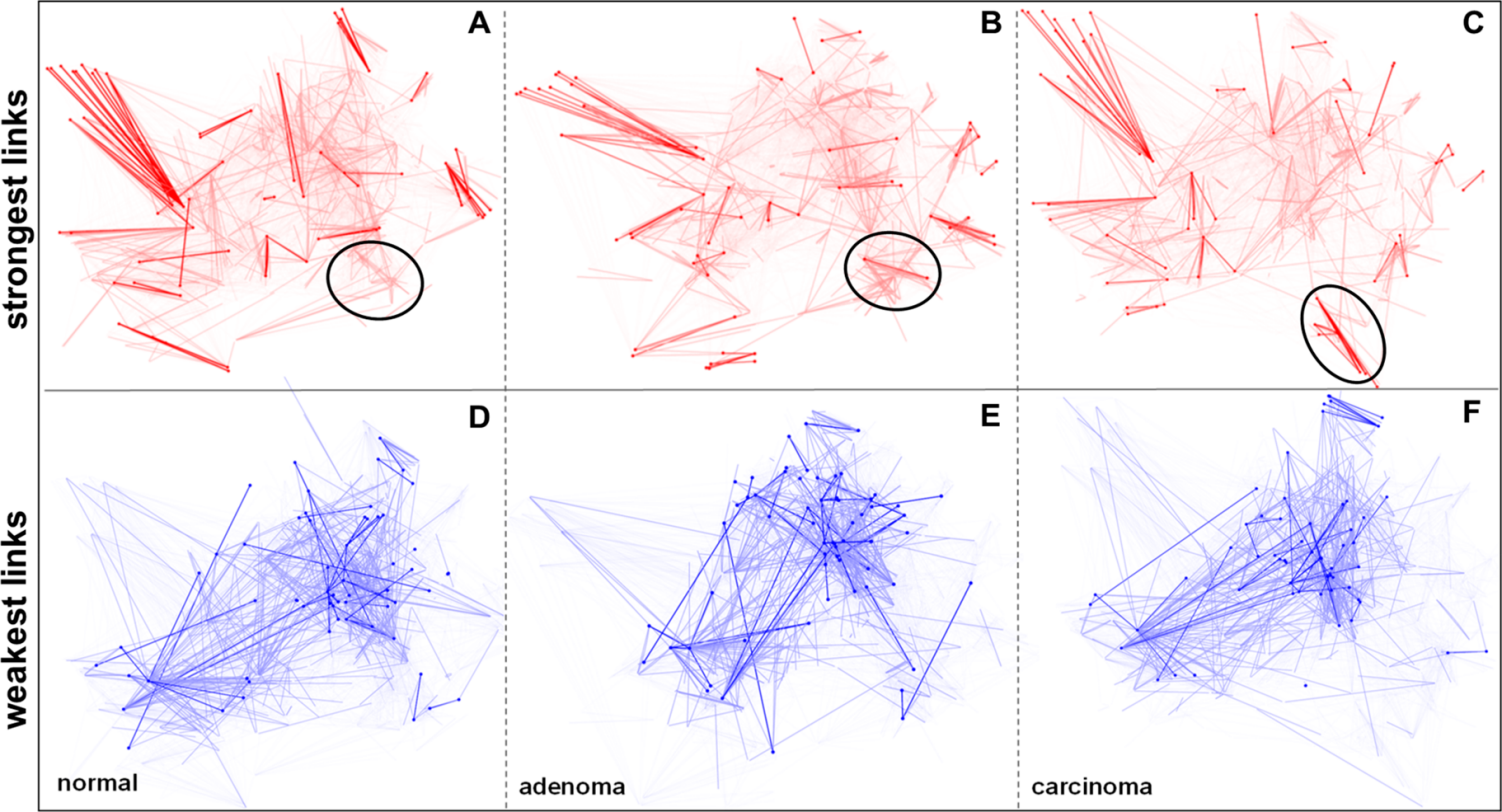
Strongest and weakest links of normal (A, D), adenoma (B, E), and carcinoma (C, F) colon data applied to the Human Cancer Signaling Network. The network was visualized using the EntOpt Cytoscape plugin.^43^ For more details, see Tables S5 and S6 (panels A–C). The top 1% of the strongest links are highlighted in red, while the brighter red lines show the top 2 to 10%. The rounded part shows the most relevant strengthening area in the adenoma and carcinoma networks, banding together as the CDK1 module shown in Figure 3 (panels D–F). The bottom 1% of the weakest links are highlighted with blue, while the brighter blue lines show the bottom 2 to 10%.

The process of carcinogenesis entails the strengthening and weakening of links according to the underlying changes in the biological functions of the cell. The distribution of the link weights (as seen in Figure 1) provides information about the general differences between the networks highlighting the strongest and weakest links (by picking the first 10% of the highest and lowest link weight values, further separating the top 1% into a subcategory Tables S5 and S6) and focusing on their functional changes adds another point of view to this work. Among the strongest links, the most prominent change is the strengthening of the cell cycle-associated links belonging to the CDK1 module (Figure 3A–C), and this result is also robust to noise (Table S7). This is consistent with the observed modular changes in the network and is associated with the inspected constant proliferation in cancer cells. Other permanently highly expressed parts of the network are responsible for immunology-associated functions such as motility and the cytokine response. The weak links contain vegetative regulation- and neuronal transmission-associated links, which make up a remarkable part of the utilized Human Cancer Signaling Network, but in colon cells, these functions are not really highlighted (Figure 3D–F). For the same reason, the structure of these links does not change among the different states of the network. In conclusion, strong and weak link structure can promote important topological changes during carcinogenesis, and the growing importance of cell cycle regulation supports the raison d’être of cancer network analysis. Link weights proved to be more sensitive to the detection of biological changes in the network than the mRNA abundances and weighted degrees, after a comparison of the representation ratio of the cell cycle and apoptosis-related modules (Table S8).

### 3.5 Changes in Targeted Therapy and Immune Checkpoint Inhibitor Associated Pathways

Targeted therapies represent a new and innovative approach in oncology. By the targeted inhibition of different signaling pathways, survival and the quality of life of metastatic colon cancer patients can be largely increased. In this research, the signaling pathways of two widely used therapeutic approaches in colon cancer, EGFR and VEGFR inhibition, and the effectiveness of immune checkpoint inhibition are estimated in advance to facilitate therapeutic decision making by examining microsatellite instability, and the loss of expression of the mismatch repair proteins responsible for it was examined by analyzing the changes in the gene expression profile of the proteins associated with the appropriate Gene Ontology terms. In addition, the effectiveness of immune checkpoint inhibition is estimated in advance to facilitate therapeutic decision making by examining microsatellite instability and the loss of expression of the mismatch repair proteins responsible for it. The mRNA expression of these proteins was also analyzed in this study by using the relevant gene ontology term (Text S4 and Table S9). Eventually, about half of the proteins associated with EGFR and VEGFR signaling and only a quarter of the proteins in the mismatch repair pathway were found in our network. The reason for this could be that the Human Cancer Signaling Network mainly contains proteins associated with signaling, thus not many proteins in the mismatch repair pathway are included.

To examining the differences among normal, adenoma, and carcinoma networks, the median abundance of each of the pathways was calculated, and for the verification of the significance of the results, paired Wilcoxon tests were conducted, as the data could have not been considered to be normally distributed (Materials and Methods and Table 2). The EGF receptor signaling was shown to be very important in the normal network, which may be because the EGF is known to be an important mediator in the alimentary tract.^49^ In the adenoma network, there is a significant reduction in its importance, showing that it is not the EGFR signaling that drives this pathologic transition.^50^ In the carcinoma network, the median abundance is almost the same as in the normal network, indicating a significant difference between the adenoma and carcinoma networks. The decreasing EGF receptor mRNA expression in our data (8.75 in the normal network, 8.06 in the adenoma network, and 5.62 in the carcinoma network) may be caused by the possibly remarkable ratio of BRAF mutant transcriptomic data in our data set (Materials and Methods), when the EGFR signaling pathway is constantly activated by the mutant BRAF protein.^51^ The median abundance of the VEGF receptor signaling is constantly growing among the three networks, which is significant between adenoma and carcinoma and between normal and carcinoma networks. It may be explained by that the level of VEGF is well known to be associated with cellular hypoxia, which is an important attribute of the cancer microenvironment.^52^ The proteins associated with mismatch repair have the largest median abundance in the adenoma network, and it is slightly decreasing between adenoma and carcinoma networks. However, these changes are not significant, which may be explained by the selection criteria for transcriptomic data, as it mainly consists of left-sided colon cancer (further details in Materials and Methods).

**Table 2:** Analysis of Targeted and Immunotherapy Pathways

	no. of associated proteins in gene ontology^a^	n umber of proteins in the network^b^	median normal abundance^c^	median adenoma abundance^c^	median carcinoma abundance^c^	*p* value (normal-adenoma)^d^	*p* value (adenoma-carcinoma)^d^	*p* value (normal-carcinoma)^d^
EGFR signaling	121	58	7.061	6.665	7.058	0.01783*	0.018*	0.67023
VEGFR signaling	93	49	6.662	7.383	7.608	0.84231	<0.001*	0.01791*
mismatch repair	38	8	8.533	9.931	8.987	0.08006	1.000	0.23395

a Number of human protein search results for GO terms: epidermal growth factor receptor signaling pathway, vascular endothelial growth factor receptor signaling pathway, and mismatch repair.

b Number of proteins in the Human Cancer Signaling Network, constructed as described in the Materials and Methods.

c Abundances were calculated from GEO data sets. For further details, see the Materials and Methods. We used median values because the data set distribution, based on Shapiro-Wilk tests, were not considered a normal distribution.

d On the basis of the results of the normality tests, we used paired Wilcoxon tests to evaluate the significance of the changes in the targeted therapy-associated data.

For further characterization of the targeted- and immunotherapy-associated nodes, median weighted degrees were calculated for each pathway. In the case of VEGFR and EGFR pathways, the median weighted degrees were more than twice those of the whole networks but not of the mismatch repair-related nodes (Table S10). The modular affiliation of each node in each network was also determined. The majority of the nodes belong to the RAC1 module, which is the largest module of the network, with non-definitively ascertainable biological functions. As a consequence, these pathways do not form distinctive modules in the network probably because they are so intertwined with every part of the graph that they cannot be separated by the modularization program (Figures S20–S22).

Links of the targeted- and immunotherapy-associated nodes can be found among the strongest and weakest links in the network with altering dynamics among the three states. The number of EGFR signaling-associated links among the top and bottom 10% of the links in the order of link weights is decreasing for the normal network (top, 42; bottom, 38), adenoma network (top, 27; bottom, 36), and carcinoma network (top, 26; bottom, 32). In VEGFR signaling, the number of links in the top 10% of the links is 63 in the normal network, 52 in the adenoma network, and 73 in the carcinoma network. The number of links in the bottom 10% of the links is steadily decreasing. In terms of the mismatch repair, there are not enough proteins in the network to see a big difference, but the decreasing expression of the pathway between the adenoma and carcinoma networks may indicate increased microsatellite instability. In conclusion, targeted therapy-associated pathways gain importance in the carcinoma network.

## 4 Discussion

The aim of this work was to analyze the changes in the network topological characteristics during the development of colon adenoma and adenocarcinoma. In the adenoma network, the strongest link weights get stronger and the weakest link weights get weaker, causing an increase in the standard deviation and implying the importance of a few highly expressed proteins. In the carcinoma network, the median link weights are larger than in the normal network; however, both the standard deviation and maximum link weights are decreasing, implying a more compact link weight distribution. Network diameter analysis also shows structural differences among the adenoma and carcinoma networks. The network diameter is the largest in the adenoma network and the smallest in the carcinoma network. This may indicate a strengthened shortcut system in cancer cells, which may help them to evade drug treatment. Modular reorganization and the analysis of the strongest and weakest links support the emergence of cancer hallmarks of constant proliferation and evading apoptosis in the carcinoma network since the cell cycle comes into prominence and the apoptotic modules unite. The steady strengthening of VEGFR and the relative strengthening of EGFR pathways compared to the adenoma network are particularly important results because the relevance of these pathways is clinically proven as these signaling pathways are involved in the treatment of advanced colon cancer.

The decrease in network diameter between normal and carcinoma networks was described multiple times in other previous topological studies.^10−15^ Our results showing the decrease in the network diameter between normal and carcinoma networks agree with those of several formal studies conducted on differential coexpression,^10,12^ association-type,^11^ protein–protein interaction,^14,15^ and ceRNA networks^13^ but not on signaling networks. The study by Wen et al. used a method very similar to that used in this study but with miRNA networks;^14^ namely, they mapped expression data on a pre-existing literature-based network structure. One particular study that found differences in the change in network diameter from normal to carcinoma networks between multiple types of cancer describe colon cancer as a type of cancer with decreasing diameter.^53^ The other results indicating high levels of compactness are unprecedented in the literature and may be part of a new approach to the description of the development of colon cancer at the system level. The strengthening of the cell cycle and the downregulation of apoptosis in cancer networks were also determined via multiple methods in previous studies.^17−20^ According to the global topological results, the carcinoma network seems to be closer to the normal network in the aspect of topological parameters than to the adenoma network, but with regards to the mesoscopic network parameters such as modular properties and the strongest and weakest links, signs of steady progression are detected.

The relevance of the targeted therapy pathways is apparent from the clinical trials, showing the efficacy of these treatments. The steadily increasing median abundance of the VEGFR pathway in this research is in line with the efficacy of VEGFR inhibitors, such as bevacizumab^54^ and ramucirumab^55^ in metastatic colon cancer. The similar median abundance of the EGFR pathway in the normal and the carcinoma networks requires further evaluation, but the EGF is already known to play an essential role in healthy alimentary tracts.^50^ The strengthening of the EGF receptor signaling pathway between colon adenoma and carcinoma was previously implied in the literature, as an increase in the EGFR copy number is detected^51^ and EGFR inhibitors such as cetuximab^56,57^ and panitumumab^58^ are widely used in therapy. The results with the targeted and immunotherapy-related pathways are important methodological feedback. Arriving at the same conclusions with network science as with molecular and clinical studies confirms the relevance of the methodology used in this study.

Our work paves the way for further research into different types of cancer networks and demands proof of the generality of systemic changes in carcinogenesis, such as regarding the link weight distribution and network diameter. The individual differences between cancer types which drive carcinogenesis and determine therapeutic possibilities can be assessed by exploring modular rearrangement and pathway enrichment. Precision oncotherapy is an emerging topic in oncology, and the analysis of gene expression data may play an important role in estimating the efficacy of different therapeutic choices.

## 5 Conclusions

The most relevant results of this work were that (i) the distribution of the link weights was spread by increasing the standard deviation in adenoma compared to those of the normal and carcinoma networks; (ii) the size of the weighted diameter is explicitly the largest in the adenoma network and the smallest in the carcinoma network; (iii) the modules of the apoptosis and cell cycle become reorganized in the adenoma and carcinoma networks; (iv) the strengthening of a group of links was observed in the area of the cell cycle in carcinoma; and (v) the observations regarding the pathways associated with targeted therapies are in good agreement with the current clinical knowledge. Our major, link-weight-based results are robust to noise. This work could provide a novel approach to cancer data analysis through modeling the development of colon cancer on the system level.

## Supporting Information Available

Text S1. Check of the robustness of the results by adding noise to the data; Text S2. algorithm for the generation of EntOpt images; Text S3. investigation of the modular overlap changes; Text S4. method for the examination of targeted and immunotherapy-related pathways; Fig. S1.hierarchy of modules -- normal; Fig. S2. hierarchy of modules -- adenoma; Fig. S3. hierarchy of modules -- carcinoma; Fig. S4. distribution of logarithmic link weights; Fig. S5. number of links in different bins of data; Fig. S6. probability density function of the logarithmic link weights; Fig. S7. cumulative distribution of the nonlogarithmic link weights; Fig. S8. distribution of protein abundances; Fig. S9. distribution of weighted degrees; Fig. S10. distribution of link weights with noise; Fig. S11. box plot of the nonlogarithmic link weights with noise; Fig. S12. EntOpt images of the unweighted Human Cancer Signaling Network; Fig. S13. EntOpt image of the network with normal weights; Fig. S14. EntOpt image of the network with adenoma weights; Fig. S15. EntOpt image of the network with carcinoma weights; Fig. S16. changes in the ModuLand overlap values based on the different number of modules with logarithmic link weights; Fig. S17. changes in the ModuLand overlap values based on the different number of modules with nonlogarithmic link weights; Fig. S18. cumulative distribution of the effective degree of modules; Fig. S19. cumulative distribution of the normalized modular overlap; Fig. S20. EGFR and VEGFR signaling and mismatch repair-related nodes in the normal network; Fig. S21. EGFR- VEGFR-signaling and mismatch repair nodes -- the adenoma network; Fig. S22. EGFR- VEGFR-signaling and mismatch repair nodes -- carcinoma network; Table S1. number of samples in the data set series; Table S2. network diameter with additional 5% noise; Table S3. function of the largest network module nodes; Table S4. largest modules of the networks with additional 5% noise; Tables S5 and S6. relevant changes in the strongest and weakest 1% of the links and Table S7. in the strongest and weakest 1% of the links with additional 5% noise; Table S8. representation of the apoptosis- and cell cycle-related modules among the strongest and weakest 10% of the abundances; weighted degrees and the link weights; Table S9. nodes in the targeted and immunotherapy-related pathways; Table S10. median weighted degrees of targeted and immunotherapy pathway-related nodes; Table S11. calculation of the diameter for the undirected network, Table S12. the directed network, and Table S13. the mixed network (PDF)

## References

[ref1] Bray F.; Ferlay J.; Soerjomataram I.; Siegel R. L.; Torre L. A.; Jemal A. Global cancer statistics 2018: GLOBOCAN estimates of incidence and mortality worldwide for 36 cancers in 185 countries. Ca-Cancer J. Clin. 2018, 68 (6), 394–424. 10.3322/caac.21492. 30207593

[ref2] Grady W. M.; Markowitz S. D. The molecular pathogenesis of colorectal cancer and its potential application to colorectal cancer screening. Dig. Dis. Sci. 2015, 60 (3), 762–72. 10.1007/s10620-014-3444-4. 25492499 PMC4779895

[ref3] Fearon E. R.; Vogelstein B. A genetic model for colorectal tumorigenesis. Cell 1990, 61 (5), 759–67. 10.1016/0092-8674(90)90186-I. 2188735

[ref4] Bardhan K.; Liu K. Epigenetics and colorectal cancer pathogenesis. Cancers 2013, 5 (2), 676–713. 10.3390/cancers5020676. 24216997 PMC3730326

[ref5] Ciombor K. K.; Bekaii-Saab T. A Comprehensive Review of Sequencing and Combination Strategies of Targeted Agents in Metastatic Colorectal Cancer. Oncologist 2018, 23 (1), 25–34. 10.1634/theoncologist.2017-0203. 29021377 PMC5759820

[ref6] Mendik P.; Dobronyi L.; Hari F.; Kerepesi C.; Maia-Moco L.; Buszlai D.; Csermely P.; Veres D. V. Translocatome: a novel resource for the analysis of protein translocation between cellular organelles. Nucleic Acids Res. 2019, 47 (D1), D495–D505. 10.1093/nar/gky1044. 30380112 PMC6324082

[ref7] Schadt E. E.; Bjorkegren J. L. NEW: network-enabled wisdom in biology, medicine, and health care. Sci. Transl. Med. 2012, 4 (115), 115rv1 10.1126/scitranslmed.3002132. 22218693

[ref8] Gosak M.; Markovic R.; Dolensek J.; Slak Rupnik M.; Marhl M.; Stozer A.; Perc M. Network science of biological systems at different scales: A review. Phys. Life Rev. 2018, 24, 118–135. 10.1016/j.plrev.2017.11.003. 29150402

[ref9] Csermely P.; Korcsmaros T.; Kiss H. J.; London G.; Nussinov R. Structure and dynamics of molecular networks: a novel paradigm of drug discovery: a comprehensive review. Pharmacol. Ther. 2013, 138 (3), 333–408. 10.1016/j.pharmthera.2013.01.016. 23384594 PMC3647006

[ref10] Deng S. P.; Zhu L.; Huang D. S. Predicting Hub Genes Associated with Cervical Cancer through Gene Co-Expression Networks. IEEE/ACM Trans. Comput. Biol. Bioinf. 2016, 13 (1), 27–35. 10.1109/TCBB.2015.2476790. 26415208

[ref11] Lee Y. S.; Hwang S. G.; Kim J. K.; Park T. H.; Kim Y. R.; Myeong H. S.; Kwon K.; Jang C. S.; Noh Y. H.; Kim S. Y. Topological network analysis of differentially expressed genes in cancer cells with acquired gefitinib resistance. Cancer Genomics Proteomics 2015, 12 (3), 153–166. 25977174

[ref12] Kugler K. G.; Mueller L. A.; Graber A.; Dehmer M. Integrative network biology: graph prototyping for co-expression cancer networks. PLoS One 2011, 6 (7), e22843 10.1371/journal.pone.0022843. 21829532 PMC3146497

[ref13] Zhou M.; Wang X.; Shi H.; Cheng L.; Wang Z.; Zhao H.; Yang L.; Sun J. Characterization of long non-coding RNA-associated ceRNA network to reveal potential prognostic lncRNA biomarkers in human ovarian cancer. Oncotarget 2016, 7 (11), 12598–611. 10.18632/oncotarget.7181. 26863568 PMC4914307

[ref14] Wen J.; Hall B.; Shi X. A network view of microRNA and gene interactions in different pathological stages of colon cancer. BMC Med. Genomics 2019, 12 (Suppl 7), 158 10.1186/s12920-019-0597-1. 31888617 PMC6936140

[ref15] Verma Y.; Yadav A.; Katara P. Mining of cancer core-genes and their protein interactome using expression profiling based PPI network approach. Gene Rep 2020, 18, 100583 10.1016/j.genrep.2019.100583.

[ref16] Yang J.; Leskovec J. Overlapping Communities Explain Core-Periphery Organization of Networks. Proc. IEEE 2014, 102 (12), 1892–1902. 10.1109/JPROC.2014.2364018.

[ref17] Qi Y. W.; Qi H. W.; Liu Z. Y.; He P. Y.; Li B. Q. Bioinformatics Analysis of Key Genes and Pathways in Colorectal Cancer. J. Comput. Biol. 2019, 26 (4), 364–375. 10.1089/cmb.2018.0237. 30810359

[ref18] Wen Z. S.; Liu Z. P.; Liu Z. R.; Zhang Y.; Chen L. N. An integrated approach to identify causal network modules of complex diseases with application to colorectal cancer. J. Am. Med. Inform Assn 2013, 20 (4), 659–667. 10.1136/amiajnl-2012-001168. PMC3721155 22967703

[ref19] Liu R.; Zhang W.; Liu Z. Q.; Zhou H. H. Associating transcriptional modules with colon cancer survival through weighted gene co-expression network analysis. BMC Genomics 2017, 18, 361 10.1186/s12864-017-3761-z. 28486948 PMC5424422

[ref20] Qu X. L.; Xie R. Q.; Chen L. N.; Feng C. C.; Zhou Y. Y.; Li W.; Huang H.; Jia X.; Lv J. J.; He Y. H.; et al. Identifying colon cancer risk modules with better classification performance based on human signaling network. Genomics 2014, 104 (4), 242–248. 10.1016/j.ygeno.2013.11.002. 24239682

[ref21] Zhou X. G.; Huang X. L.; Liang S. Y.; Tang S. M.; Wu S. K.; Huang T. T.; Mo Z. N.; Wang Q. Y. Identifying miRNA and gene modules of colon cancer associated with pathological stage by weighted gene co-expression network analysis. OncoTargets Ther. 2018, 11, 2815–2830. 10.2147/OTT.S163891. PMC5961473 29844680

[ref22] Yu H. L.; Ye L.; Wang J. X.; Jin L.; Lv Y. F.; Yu M. Protein-protein interaction networks and modules analysis for colorectal cancer and serrated adenocarcinoma. J. Cancer Res. Ther 2015, 11 (4), 846–851. 10.4103/0973-1482.140805. 26881529

[ref23] Petrochilos D.; Shojaie A.; Gennari J.; Abernethy N. Using random walks to identify cancer-associated modules in expression data. BioData Min. 2013, 6, 17 10.1186/1756-0381-6-17. 24128261 PMC4015830

[ref24] Csermely P.; Korcsmaros T.; Nussinov R. Intracellular and intercellular signaling networks in cancer initiation, development and precision anti-cancer therapy RAS acts as contextual signaling hub. Semin. Cell Dev. Biol. 2016, 58, 55–59. 10.1016/j.semcdb.2016.07.005. 27395026 PMC5028272

[ref25] Nussinov R.; Tsai C. J.; Jang H.; Korcsmaros T.; Csermely P. Oncogenic KRAS signaling and YAP1/beta-catenin: Similar cell cycle control in tumor initiation. Semin. Cell Dev. Biol. 2016, 58, 79–85. 10.1016/j.semcdb.2016.04.001. 27058752

[ref26] Nussinov R.; Tsai C. J.; Csermely P. Allo-network drugs: harnessing allostery in cellular networks. Trends Pharmacol. Sci. 2011, 32 (12), 686–93. 10.1016/j.tips.2011.08.004. 21925743 PMC7380718

[ref27] Cui Q.; Ma Y.; Jaramillo M.; Bari H.; Awan A.; Yang S.; Zhang S.; Liu L.; Lu M.; O’Connor-McCourt M.; et al. A map of human cancer signaling. Mol. Syst. Biol. 2007, 3, 152 10.1038/msb4100200. 18091723 PMC2174632

[ref28] Kovacs I. A.; Palotai R.; Szalay M. S.; Csermely P. Community landscapes: an integrative approach to determine overlapping network module hierarchy, identify key nodes and predict network dynamics. PLoS One 2010, 5 (9), e12528 10.1371/journal.pone.0012528. 20824084 PMC2932713

[ref29] Ashburner M.; Ball C. A.; Blake J. A.; Botstein D.; Butler H.; Cherry J. M.; Davis A. P.; Dolinski K.; Dwight S. S.; Eppig J. T.; et al. Gene ontology: tool for the unification of biology. The Gene Ontology Consortium. Nat. Genet. 2000, 25 (1), 25–9. 10.1038/75556. 10802651 PMC3037419

[ref30] Carbon S.; Douglass E.; Dunn N.; Good B.; Harris N. L.; Lewis S. E.; Mungall C. J.; Basu S.; Chisholm R. L.; Dodson R. J.; et al. The Gene Ontology Resource: 20 years and still GOing strong. Nucleic Acids Res. 2019, 47 (D1), D330–D338. 10.1093/nar/gky1055. 30395331 PMC6323945

[ref31] Tate J. G.; Bamford S.; Jubb H. C.; Sondka Z.; Beare D. M.; Bindal N.; Boutselakis H.; Cole C. G.; Creatore C.; Dawson E.; et al. COSMIC: the Catalogue Of Somatic Mutations In Cancer. Nucleic Acids Res. 2019, 47 (D1), D941–D947. 10.1093/nar/gky1015. 30371878 PMC6323903

[ref32] Nishimura D. BioCarta. Biotech Software & Internet Report: The Computer Software Journal for Scient 2001, 2 ( (3), ), 117–120.

[ref33] Skrzypczak M.; Goryca K.; Rubel T.; Paziewska A.; Mikula M.; Jarosz D.; Pachlewski J.; Oledzki J.; Ostrowski J. Modeling oncogenic signaling in colon tumors by multidirectional analyses of microarray data directed for maximization of analytical reliability. PLoS One 2010, 5 (10), e13091 10.1371/journal.pone.0013091. 20957034 PMC2948500

[ref34] Galamb O.; Gyorffy B.; Sipos F.; Spisak S.; Nemeth A. M.; Miheller P.; Tulassay Z.; Dinya E.; Molnar B. Inflammation, adenoma and cancer: objective classification of colon biopsy specimens with gene expression signature. Dis. Markers 2008, 25 (1), 586721 10.1155/2008/586721. PMC3827801 18776587

[ref35] Sabates-Bellver J.; Van der Flier L. G.; de Palo M.; Cattaneo E.; Maake C.; Rehrauer H.; Laczko E.; Kurowski M. A.; Bujnicki J. M.; Menigatti M.; et al. Transcriptome profile of human colorectal adenomas. Mol. Cancer Res. 2007, 5 (12), 1263–75. 10.1158/1541-7786.MCR-07-0267. 18171984

[ref36] Valcz G.; Patai A. V.; Kalmar A.; Peterfia B.; Furi I.; Wichmann B.; Muzes G.; Sipos F.; Krenacs T.; Mihaly E.; et al. Myofibroblast-Derived SFRP1 as Potential Inhibitor of Colorectal Carcinoma Field Effect. PLoS One 2014, 9 (11), e106143 10.1371/journal.pone.0106143. 25405986 PMC4236006

[ref37] de Sousa E Melo F.; Colak S.; Buikhuisen J.; Koster J.; Cameron K.; de Jong J. H.; Tuynman J. B.; Prasetyanti P. R.; Fessler E.; van den Bergh S. P.; Rodermond H.; Dekker E.; van der Loos C. M.; Pals S. T.; van de Vijver M. J.; Versteeg R.; Richel D. J.; Vermeulen L.; Medema J. P.; et al. Methylation of cancer-stem-cell-associated Wnt target genes predicts poor prognosis in colorectal cancer patients. Cell Stem Cell 2011, 9 (5), 476–485. 10.1016/j.stem.2011.10.008. 22056143

[ref38] Xing S.; Wallmeroth N.; Berendzen K. W.; Grefen C. Techniques for the Analysis of Protein-protein Interactions in Vivo. Plant Physiol. 2016, 171 (2), 727–758. 10.1104/pp.16.00470. 27208310 PMC4902627

[ref39] Edwards P. R.; Gill A.; Pollardknight D. V.; Hoare M.; Buckle P. E.; Lowe P. A.; Leatherbarrow R. J. Kinetics of Protein-protein Interactions at the Surface of an Optical Biosensor. Anal. Biochem. 1995, 231 (1), 210–217. 10.1006/abio.1995.1522. 8678303

[ref40] Mihalik Á.; Csermely P. Heat Shock Partially Dissociates the Overlapping Modules of the Yeast Protein-protein Interaction Network: A Systems Level Model of Adaptation. PLoS Comput. Biol. 2011, 7 (10), e1002187 10.1371/journal.pcbi.1002187. 22022244 PMC3192799

[ref41] Hagberg A.; Swart P.; Chult D. Exploring network structure, dynamics, and function using NetworkX. In Proc. 7th Python Science Conference (SciPy 2008) Varoquaux G., Vaught T., Millman J., Eds.; Los Alamos National Laboratory (LANL): Los Alamos, NM, 2008; pp 11–15.

[ref42] Dijkstra E. W. A note on two problems in connexion with graphs. Numerische mathematik 1959, 1 (1), 269–271. 10.1007/BF01386390.

[ref43] Ágg B.; Császár A.; Szalay-Bekő M.; Veres D. V.; Mizsei R.; Ferdinandy P.; Csermely P.; Kovács I. A. The EntOptLayout Cytoscape plug-in for the efficient visualization of major protein complexes in protein–protein interaction and signalling networks. Bioinformatics 2019, 35 (21), 4490–4492. 10.1093/bioinformatics/btz257. 31004478 PMC6821346

[ref44] Kovacs I. A.; Mizsei R.; Csermely P. A unified data representation theory for network visualization, ordering and coarse-graining. Sci. Rep. 2015, 5, 13786 10.1038/srep13786. 26348923 PMC4642569

[ref45] Szalay-Beko M.; Palotai R.; Szappanos B.; Kovacs I. A.; Papp B.; Csermely P. ModuLand plug-in for Cytoscape: determination of hierarchical layers of overlapping network modules and community centrality. Bioinformatics 2012, 28 (16), 2202–2204. 10.1093/bioinformatics/bts352. 22718784

[ref46] Amini H.; Lelarge M. The diameter of weighted random graphs. Annals of Applied Probability 2015, 25 (3), 1686–1727. 10.1214/14-AAP1034.

[ref47] Bateman A.; Martin M. J.; Orchard S.; Magrane M.; Alpi E.; Bely B.; Bingley M.; Britto R.; Bursteinas B.; et al. UniProt: a worldwide hub of protein knowledge. Nucleic Acids Res. 2019, 47 (D1), D506–D515. 10.1093/nar/gky1049. 30395287 PMC6323992

[ref48] Waldman T.; Kinzler K. W.; Vogelstein B. p21 is necessary for the p53-mediated G1 arrest in human cancer cells. Cancer Res. 1995, 55 (22), 5187–5190. 7585571

[ref49] Tang X.; Liu H.; Yang S.; Li Z.; Zhong J.; Fang R. Epidermal Growth Factor and Intestinal Barrier Function. Mediators Inflammation 2016, 2016, 1927348 10.1155/2016/1927348. PMC4976184 27524860

[ref50] Flora M.; Piana S.; Bassano C.; Bisagni A.; De Marco L.; Ciarrocchi A.; Tagliavini E.; Gardini G.; Tamagnini I.; Banzi C.; et al. Epidermal growth factor receptor (EGFR) gene copy number in colorectal adenoma-carcinoma progression. Cancer Genet. 2012, 205 (12), 630–5. 10.1016/j.cancergen.2012.10.005. 23181982

[ref51] Corcoran R. B.; Ebi H.; Turke A. B.; Coffee E. M.; Nishino M.; Cogdill A. P.; Brown R. D.; Della Pelle P.; Dias-Santagata D.; Hung K. E.; et al. EGFR-Mediated Reactivation of MAPK Signaling Contributes to Insensitivity of BRAF-Mutant Colorectal Cancers to RAF Inhibition with Vemurafenib. Cancer Discovery 2012, 2 (3), 227–235. 10.1158/2159-8290.CD-11-0341. 22448344 PMC3308191

[ref52] Mizukami Y.; Li J. N.; Zhang X. B.; Zimmer M. A.; Iliopoulos O.; Chung D. C. Hypoxia-inducible factor-1-independent regulation of vascular endothelial growth factor by hypoxia in colon cancer. Cancer Res. 2004, 64 (5), 1765–1772. 10.1158/0008-5472.CAN-03-3017. 14996738

[ref53] Rahman K. T.; Islam M. F.; Banik R. S.; Honi U.; Diba F. S.; Sumi S. S.; Kabir S. M. T.; Akhter M. S. Changes in protein interaction networks between normal and cancer conditions: Total chaos or ordered disorder?. Network Biology 2013, 3 (1), 15.

[ref54] Rosen L. S.; Jacobs I. A.; Burkes R. L. Bevacizumab in Colorectal Cancer: Current Role in Treatment and the Potential of Biosimilars. Target Oncol 2017, 12 (5), 599–610. 10.1007/s11523-017-0518-1. 28801849 PMC5610666

[ref55] Verdaguer H.; Tabernero J.; Macarulla T. Ramucirumab in metastatic colorectal cancer: evidence to date and place in therapy. Ther. Adv. Med. Oncol. 2016, 8 (3), 230–242. 10.1177/1758834016635888. 27239240 PMC4872251

[ref56] Guren T. K.; Thomsen M.; Kure E. H.; Sorbye H.; Glimelius B.; Pfeiffer P.; Osterlund P.; Sigurdsson F.; Lothe I. M. B.; Dalsgaard A. M.; et al. Cetuximab in treatment of metastatic colorectal cancer: final survival analyses and extended RAS data from the NORDIC-VII study. Br. J. Cancer 2017, 116 (10), 1271–1278. 10.1038/bjc.2017.93. 28399112 PMC5482736

[ref57] Van Cutsem E.; Kohne C. H.; Hitre E.; Zaluski J.; Chien C. R. C.; Makhson A.; D’Haens G.; Pinter T.; Lim R.; Bodoky G.; et al. Cetuximab and Chemotherapy as Initial Treatment for Metastatic Colorectal Cancer. N. Engl. J. Med. 2009, 360 (14), 1408–1417. 10.1056/NEJMoa0805019. 19339720

[ref58] Battaglin F.; Puccini A.; Djaballah S. A.; Lenz H. J. The impact of panitumumab treatment on survival and quality of life in patients with RAS wild-type metastatic colorectal cancer. Cancer Manage. Res. 2019, 11, 5911–5924. 10.2147/CMAR.S186042. PMC6607986 31388315

