# Supplementary material for: Modular Reorganization of Signaling Networks during the Development of Colon Adenoma and Carcinoma
Source: J Phys Chem B. 2021 Feb 9;125(7):1716–26. doi: 10.1021/acs.jpcb.0c09307 (PMC8023713; doi:10.1021/acs.jpcb.0c09307)
Supplement: Supplementary file 1 — jp0c09307_si_002.pdf [file jp0c09307_si_002.pdf]

Supporting Information for Publication

Modular Reorganization of Signaling Networks During the Development of Colon Adenoma and Carcinoma

Klára Schulc[1], Zsolt T. Nagy[1], Sebestyén Kamp[2], János Molnár[2], Daniel V. Veres[2], Peter Csermely[1], Borbála M. Kovács[1]

[1]Department of Molecular Biology, Semmelweis University, Budapest 1428, Hungary

[2]Turbine AI, Budapest, Hungary

Table of Contents

Supporting Texts 5

Text S1. Checking the robustness of the results by adding noise to the data 5

Text S2. The algorithm for the generation of EntOpt images 6

Text S3. Investigating into the modular overlap changes 7

Text S4. The method for the examination of targeted and immunotherapy-related pathways 8

Supporting Figures.. 9

Figure S1. The hierarchy of modules in Level 1, calculated with the ModuLand plugin of Cytoscape, with normal link weights...................... 9

Figure S2. The hierarchy of modules in Level 1, calculated with the ModuLand plugin of Cytoscape, with adenoma link weights................. 10

Figure S3. The hierarchy of modules in Level 1, calculated with the ModuLand plugin of Cytoscape, with carcinoma link weights.............. 11

Figure S4. The distribution of the logarithmic link weights.................... 12

Figure S5. Number of links in different bins of data 13

Figure S6. Probability density function of the logarithmic link weights 14

Figure S7. Cumulative distribution and box plot of the non-logarithmic link weights 15

Figure S8. Cumulative distribution of the abundances.......................... 17

Figure S9. Cumulative distribution of weighted degrees....................... 18

Figure S10. Cumulative distribution of link weights with additional 5% noise 19

Figure S11. Box plot of link weights with additional 5% noise.............. 20

Figure S12. The EntOpt image of the unweighted Human Cancer Signaling Network 21

Figure S13. The EntOpt image of the network with normal weights...... 22

Figure S14. The EntOpt image of the network with adenoma weights .. 23

Figure S15. The EntOpt image of the network with carcinoma weights 24

Figure S16. Change of the ModuLand overlap values based on the different number of modules with logarithmic link weights 25

Figure S17. Change of the ModuLand overlap values based on the different number of modules with non-logarithmic link weights 26

Figure S18. Cumulative distribution of the effective degree of modules 27

Figure S19. Cumulative distribution of the normalized modular overlap 28

Figure S20. EGFR-, VEGFR-signaling and mismatch repair related nodes in the normal network.. 29

Figure S21. EGFR-, VEGFR-signaling and mismatch repair related nodes in the adenoma network.. 30

Figure S22. EGFR-, VEGFR-signaling and mismatch repair related nodes in the carcinoma network.. 31

Supporting Tables 32

Table S1. Number of samples in the dataset series 32

Table S2. Network diameter calculations with reciprocal and inverted data, and with additional 5% noise... 33

Table S3. Function of the largest network modules nodes 34

Table S4. The largest modules of the networks with additional 5% noise 36

Tables S5 and S6. The relevant changes in the strongest and weakest 1% of the links... 37

Table S7. The relevant changes in the strongest and weakest 1% of the links with additional 5% noise 42

Table S8. Representation of the apoptosis and cell cycle related modules among strongest and weakest 10% of the abundances, weighted degrees and the link weights.. 44

Table S9. The nodes in the targeted and immunotherapy related pathways 46

Table S10. Median weighted degrees of targeted and immunotherapy pathway related nodes 49

Supporting Codes.. 50

Code S1. Calculating diameter for undirected network 50

Code S2. Calculating diameter for directed network 55

Code S3. Calculating diameter for mixed network.................................. 60

Supporting Texts

Text S1. Checking the robustness of the results by adding noise to the data

Microarray data is well known to be often noisy. To improve the quality we collected data over 100 colon samples from each state (normal; adenoma; carcinoma). We chose to analyze parameters that do not rely and require precise measurements. However, we investigated into the robustness of our results by randomly elevating and decreasing half-half of the abundances by 5%. We found that our most relevant results are robust to this noise (see Figures S10 and S11 Tables S2 and S4).

Text S2. The algorithm for the generation of EntOpt images

Entropy-based visualisation (http://apps.cytoscape.org/apps/entoptlayout) was extensively used in this research to visualise the important changes among the normal, adenoma and carcinoma networks with a link weight sensitive method. The algorithm for the optimal usage of the EntOpt Layout program was described by a member of our research group, Andrea Császár.

i. Each network was first visualised by the built-in Prefuse Force Directed Layout program of Cytoscape.

ii. The maximal run time was set to be 50 000 seconds.

iii. Finally, the program was runned four times, with different settings:

1) Initialize node positions with: Visible coordinates

   Node parameter to optimize for: Position

2) Initialize node positions with: Entopt coordinates

   Node parameter to optimize for: Width

3) Initialize node positions with: Entopt coordinates

   Node parameter to optimize for: Position

4) Initialize node positions with: Entopt coordinates

   Node parameter to optimize for: Width

Text S3. Investigating into the modular overlap changes

The ModuLand plugin is able to detect highly overlapping modules, as well as to calculate the effective number and degree of modules. After comparing these between the three networks, we have found that although there are interesting differences, they are equivalent to the number of modules. Therefore, after normalization to the same number of modules, the differences in the overlap values disappeared. Calculating the exact number of modules is a current challenge in network science, thus these results (Figures S16-19) are interesting, but not reliable enough.

Text S4. The method for the examination of targeted and immunotherapy-related pathways

Targeted and immunotherapy related pathways were analysed with the help of Gene Ontology Consortium, as the proteins belonging to the appropriate GO terms were selected. In case of EGFR and VEGFR inhibitors, 'epidermal growth factor receptor signaling pathway' and 'vascular endothelial growth factor receptor signaling pathway' terms were used, respectively. In the case of immunotherapy, MSI status and mismatch repair proteins are known to be important predictors of therapy efficacy. In this case, the proteins of the GO term 'mismatch repair' were chosen for analysis.

As a next step, after filtering for human proteins, the list of the proteins belonging to the appropriate GO term was downloaded, and the duplications were removed. Then the overlap with our dataset were calculated, and the abundance of the remaining proteins were looked up. Then, the median abundance was calculated for each of the pathways. Their modular affiliation and the relations with the strongest and weakest links in the network were also analysed.

Supporting Figures

Figure S1. The hierarchy of modules in Level 1, calculated with the ModuLand plugin of Cytoscape, with normal link weights

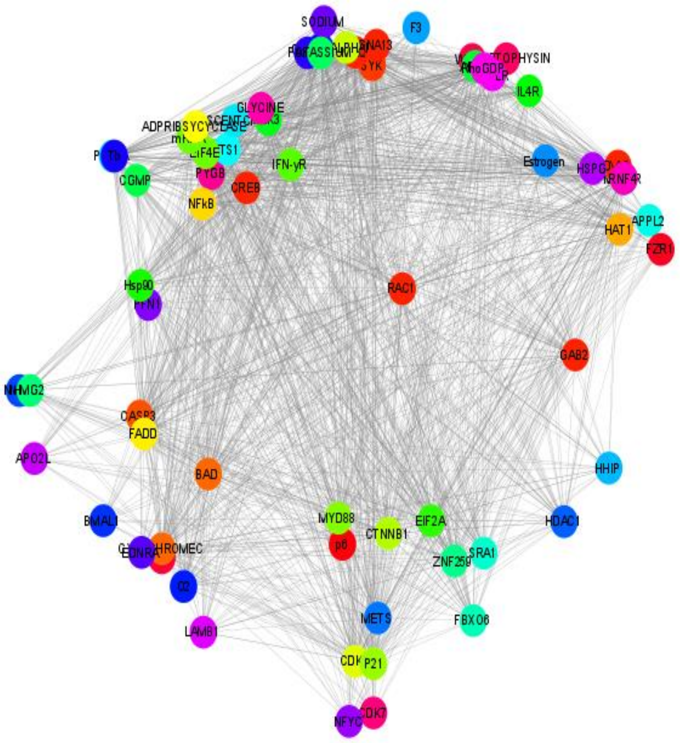

This figure was generated with the ModuLand program. After finding the functional modules in the network, the program generates a hierarchy between them, with the use of the modular link weights. The modules are highlighted with different colors, and the links between them are depicted with grey lines. Normal link weights were used in this calculation.

Figure S2. The hierarchy of modules in Level 1, calculated with the ModuLand plugin of Cytoscape, with adenoma link weights

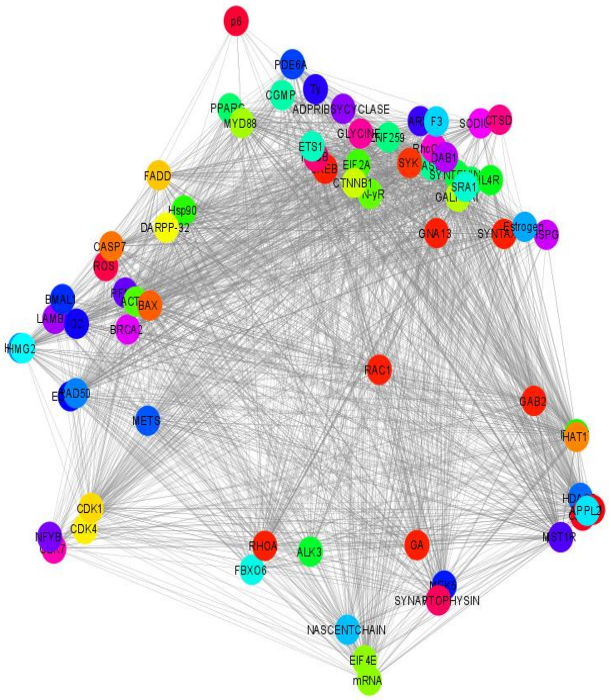

This figure was generated with the ModuLand program. After finding the functional modules in the network, the program generates a hierarchy between them, with the use of the modular link weights. The modules are highlighted with different colors, and the links between them are depicted with grey lines. Adenoma link weights were used in this calculation.

Figure S3. The hierarchy of modules in Level 1, calculated with the ModuLand plugin of Cytoscape, with carcinoma link weights

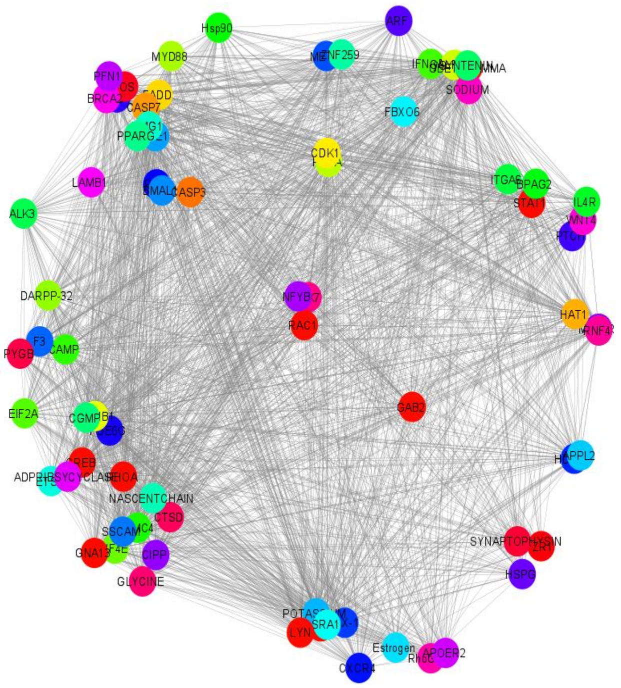

This figure was generated with the ModuLand program. After finding the functional modules in the network, the program generates a hierarchy between them, with the use of the modular link weights. The modules are highlighted with different colors, and the links between them are depicted with grey lines. Carcinoma link weights were used in this calculation.

Figure S4. The distribution of the logarithmic link weights

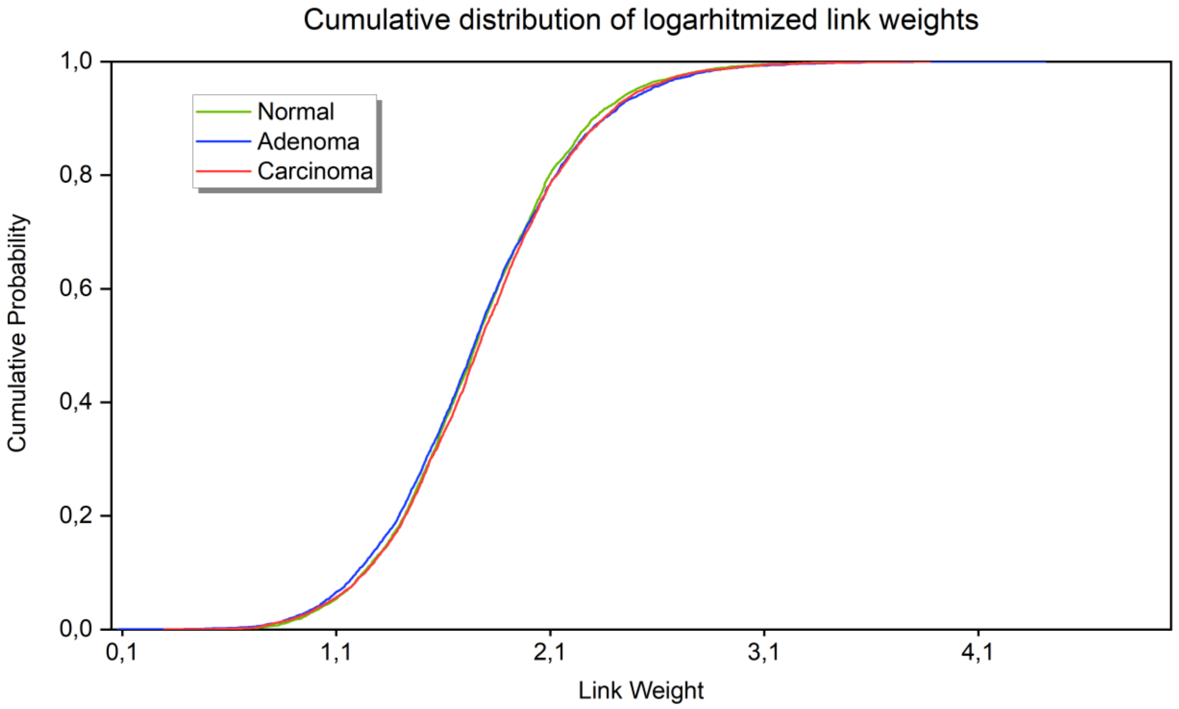

The distribution of the logarithmic link weights demonstrates the large median and standard deviation of the adenoma network, similarly to the box-plot and distribution of the logarithmic and non-logarhitmic values (see the main text).

Figure S5. Number of links in different bins of data

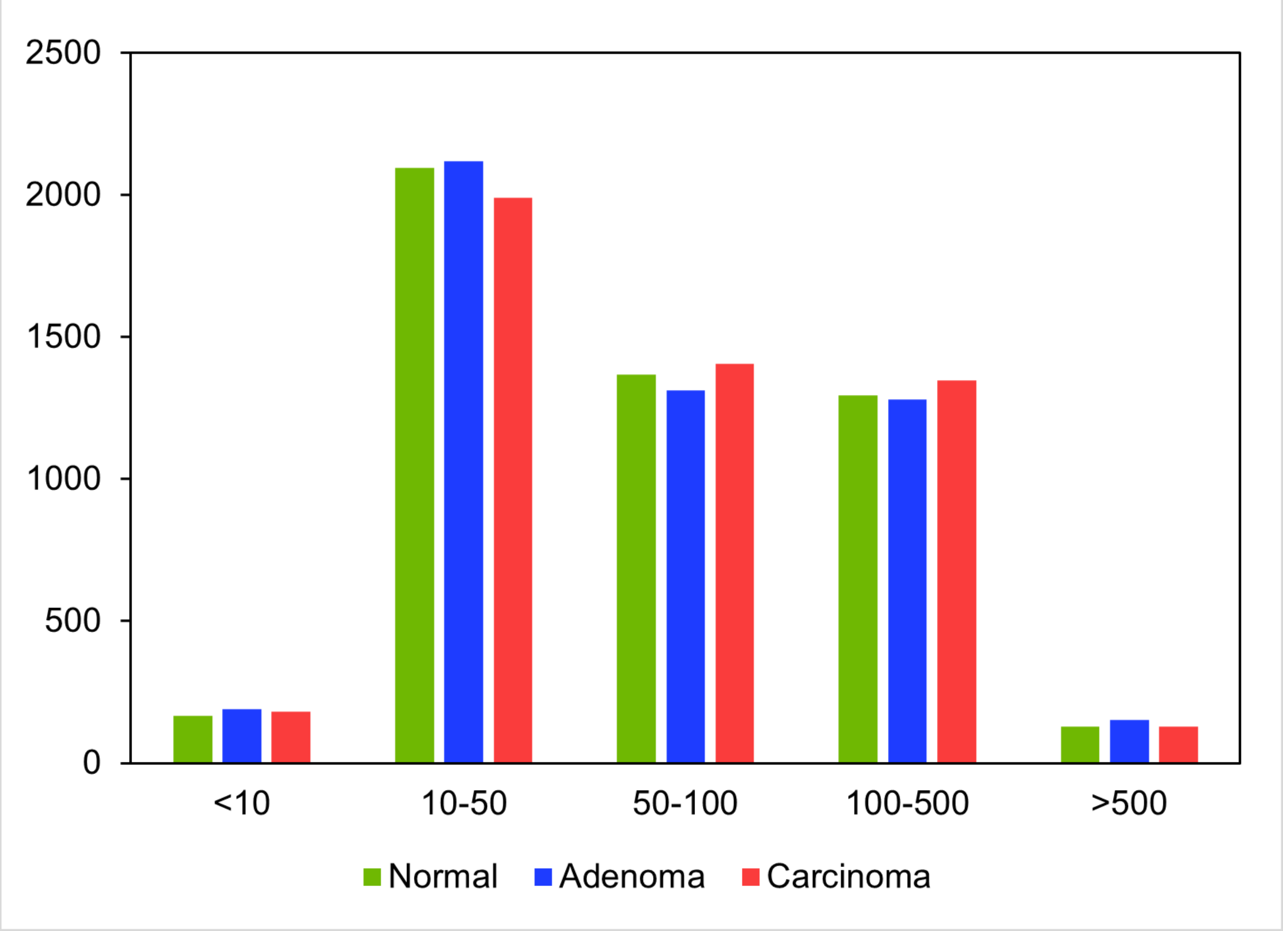

To further demonstrate the differences among the three networks' link weight distribution (see the main text and Figure S6), the link weight data was binned according to the non-logarhitmic link weight cutoff values. It also shows that the adenoma network is the most important among the very small and very large link weights.

Figure S6. Probability density function of the logarithmic link weights

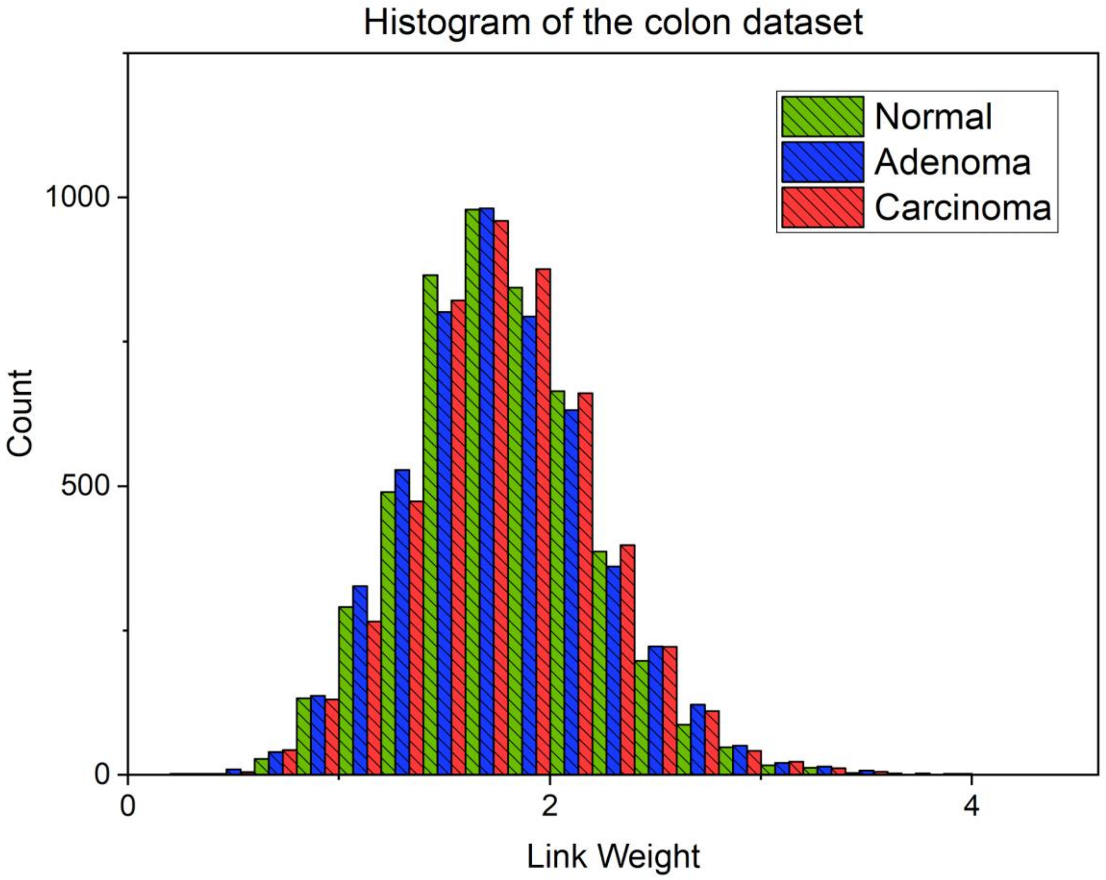

The probability density function of the logarithmic link weight data also demonstrates subtle differences between the distribution of the three networks.

Figure S7. Cumulative distribution and box plot of the non-logarithmic link weights

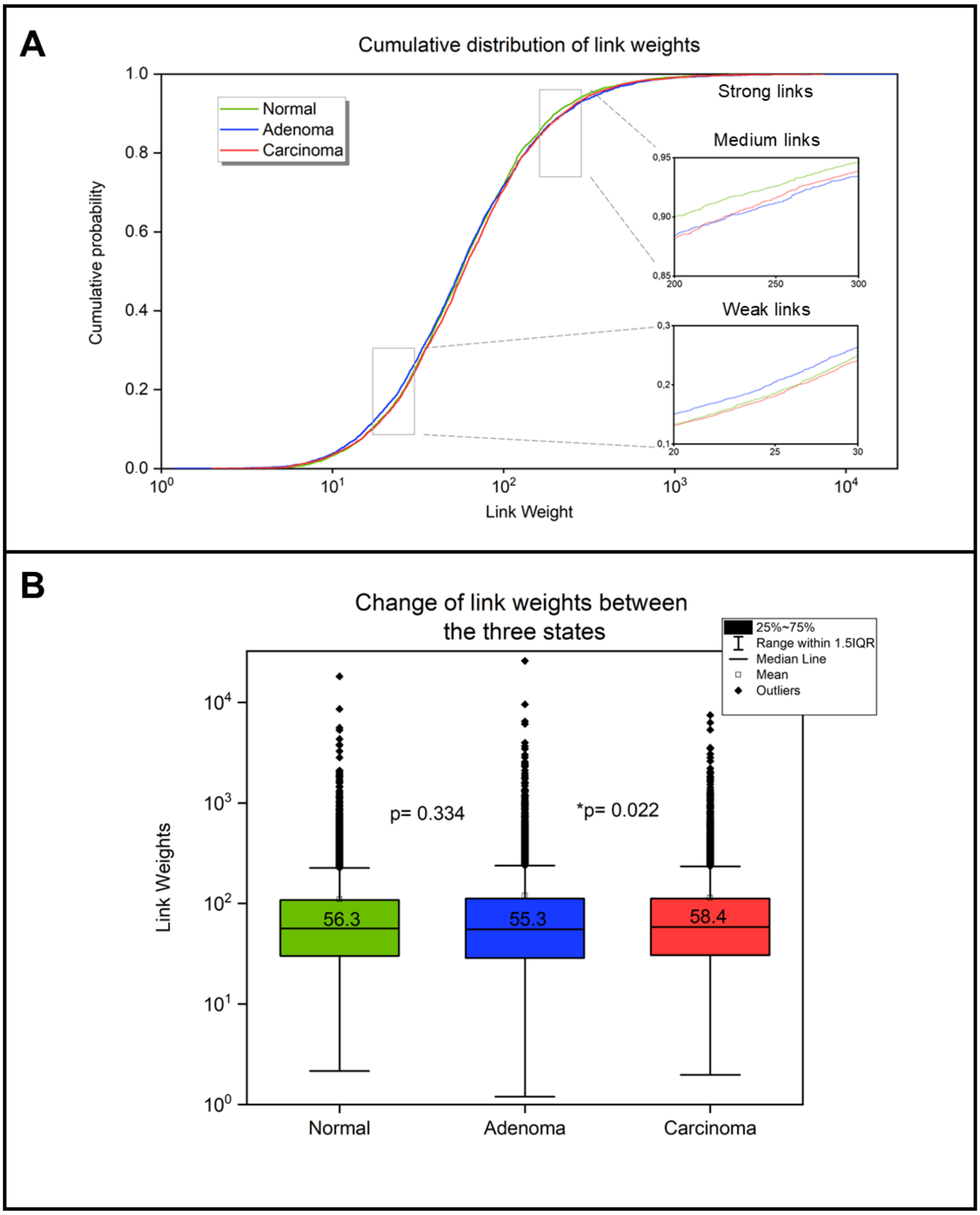

The cumulative distribution of the non-logarithmic link weights shows the same differences as with the logarithmic values (see the main text). Two areas among the small link weights (defined as link weights under 50) and medium link weights (defined as link weights between 100 and 500) are highlighted.

The box-plot of the non-logarithmic link weight demonstrates the large median and standard deviation of the adenoma network, similarly to the box-plot of the logarithmic values (see the main text). The median and p values (paired Wilcoxon) are highlighted.

Figure S8. Cumulative distribution of the abundances

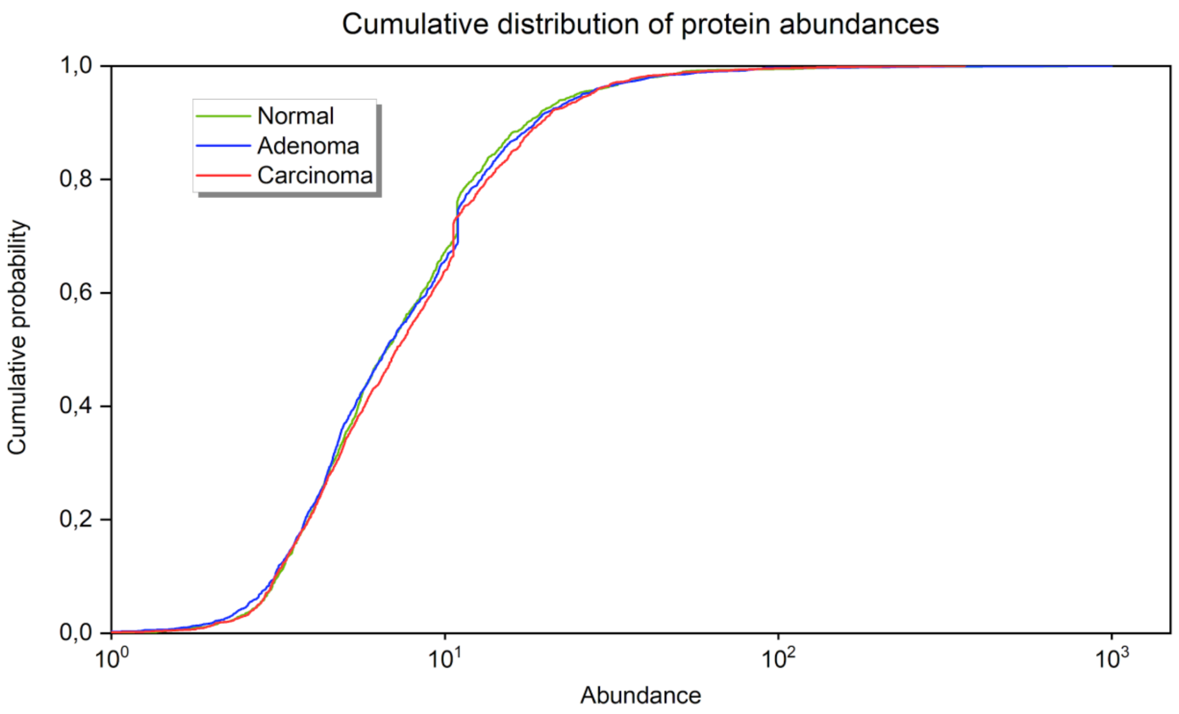

The cumulative distribution of the protein abundances also shows the same main characteristics as the link weight distribution (see the main text). As in the case of a few missing values, the average abundance has been used, a small bulge appears around the average value. This correction did not affect the analysis significantly.

Figure S9. Cumulative distribution of weighted degrees

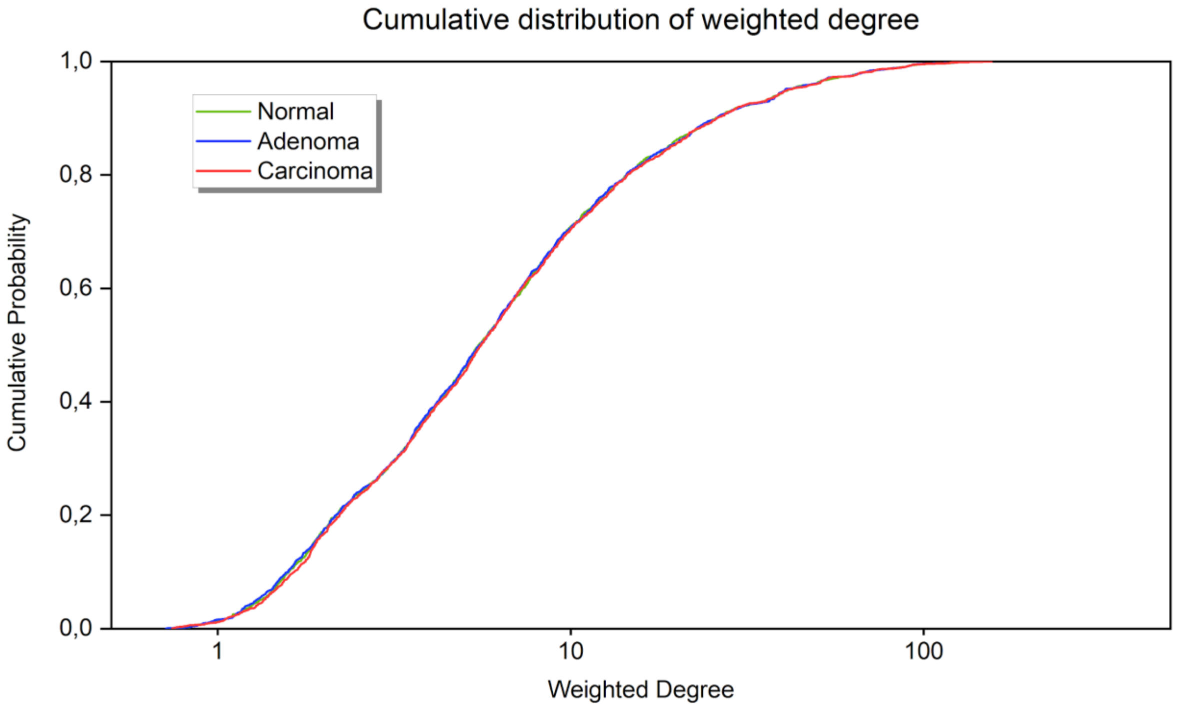

The cumulative distribution of the weighted degrees shows less demonstrable differences than the link weight and abundance distribution (see the main text and Figures S6 and S8). In the area of small and large link weights, the adenoma network has the largest cumulative probability.

Figure S10. Cumulative distribution of link weights with additional 5% noise

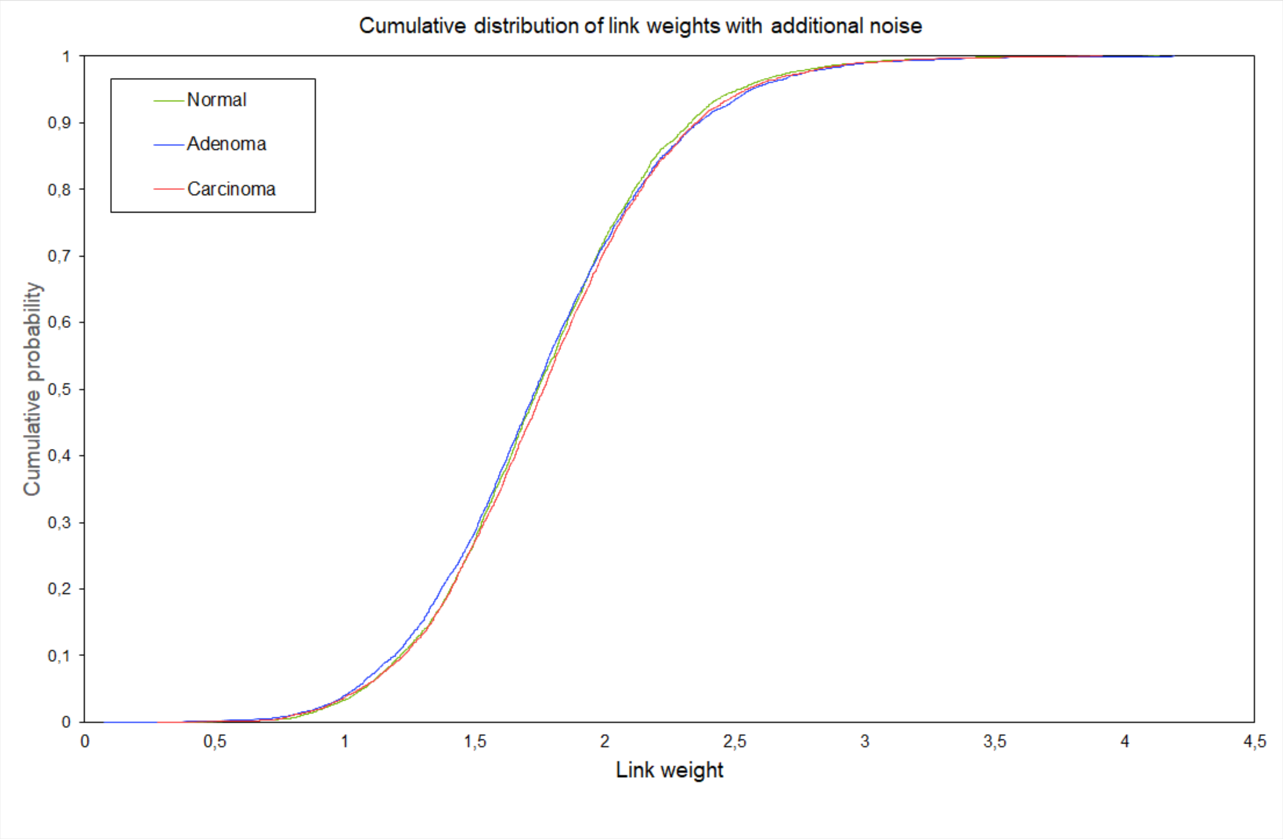

The noisy network was created as described in Text S1. The results are robust to noise, as the adenoma network has the most cumulative probability among the small link weights, and the normal network among the medium link weights (see the main text for context). The results are also significant (paired Wilcoxon-test, $p<0.0001$ for normal-adenoma, adenoma-carcinoma, normal-carcinoma pairs).

Figure S11. Box plot of link weights with additional 5% noise

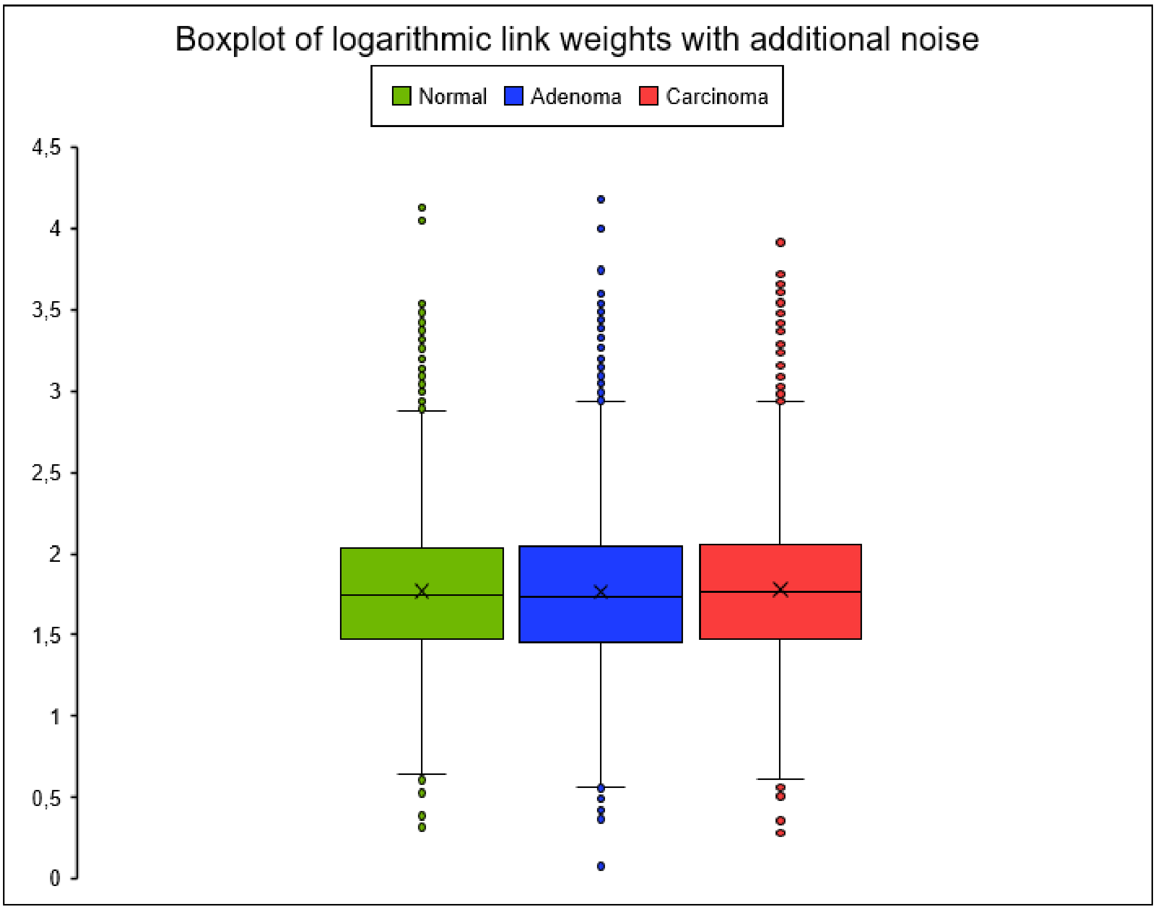

The noisy network was created as described in Text S1. The results are robust to noise, as the adenoma network has the largest, and the carcinoma network has the smallest standard deviation (see the main text for context).

Figure S12. The EntOpt image of the unweighted Human Cancer Signaling Network

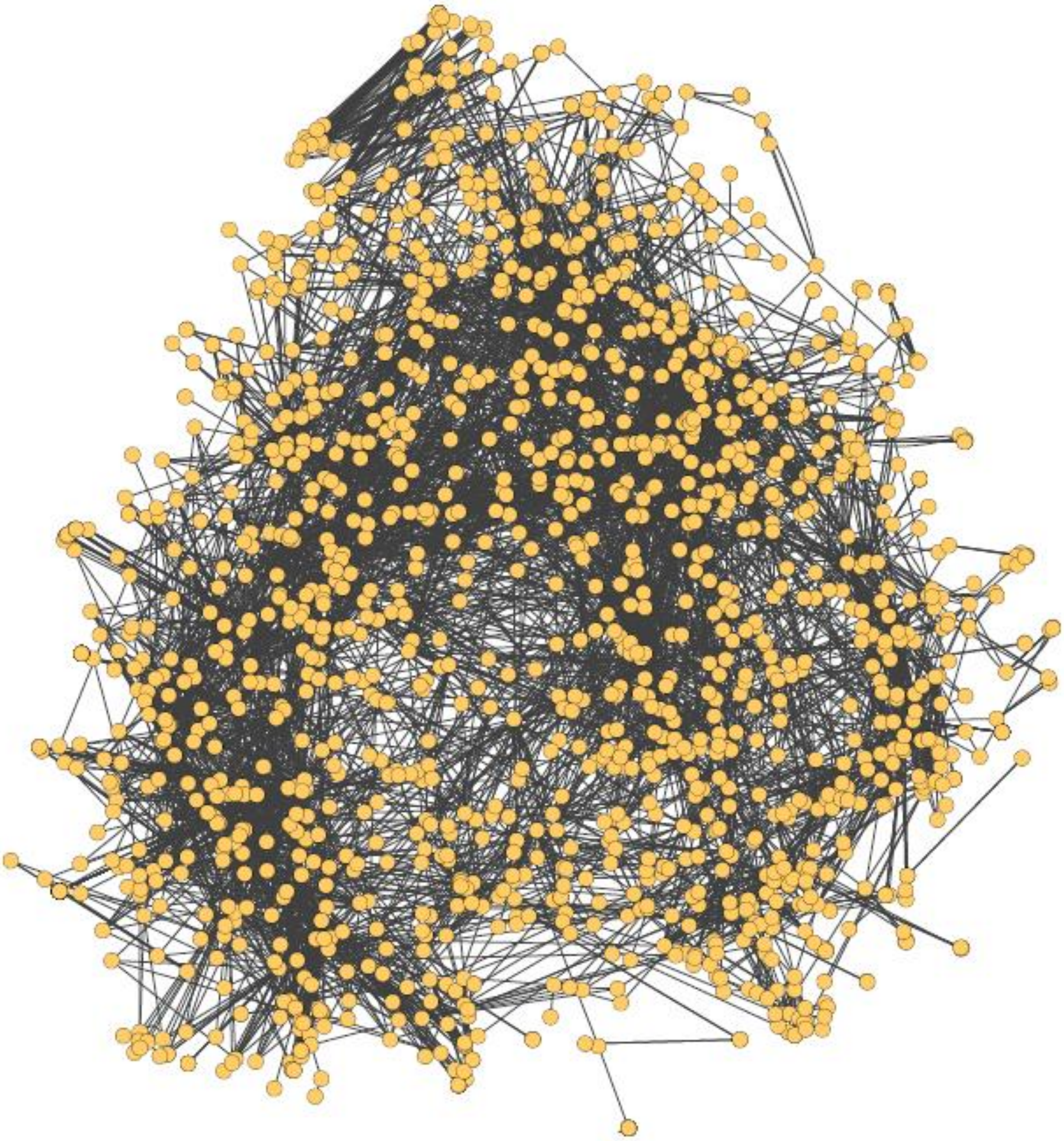

The image was made with the EntOpt Layout program, as described above. The entropy calculations were conducted without the use of link weights. The nodes are highlighted with the color yellow.

Figure S13. The EntOpt image of the network with normal weights

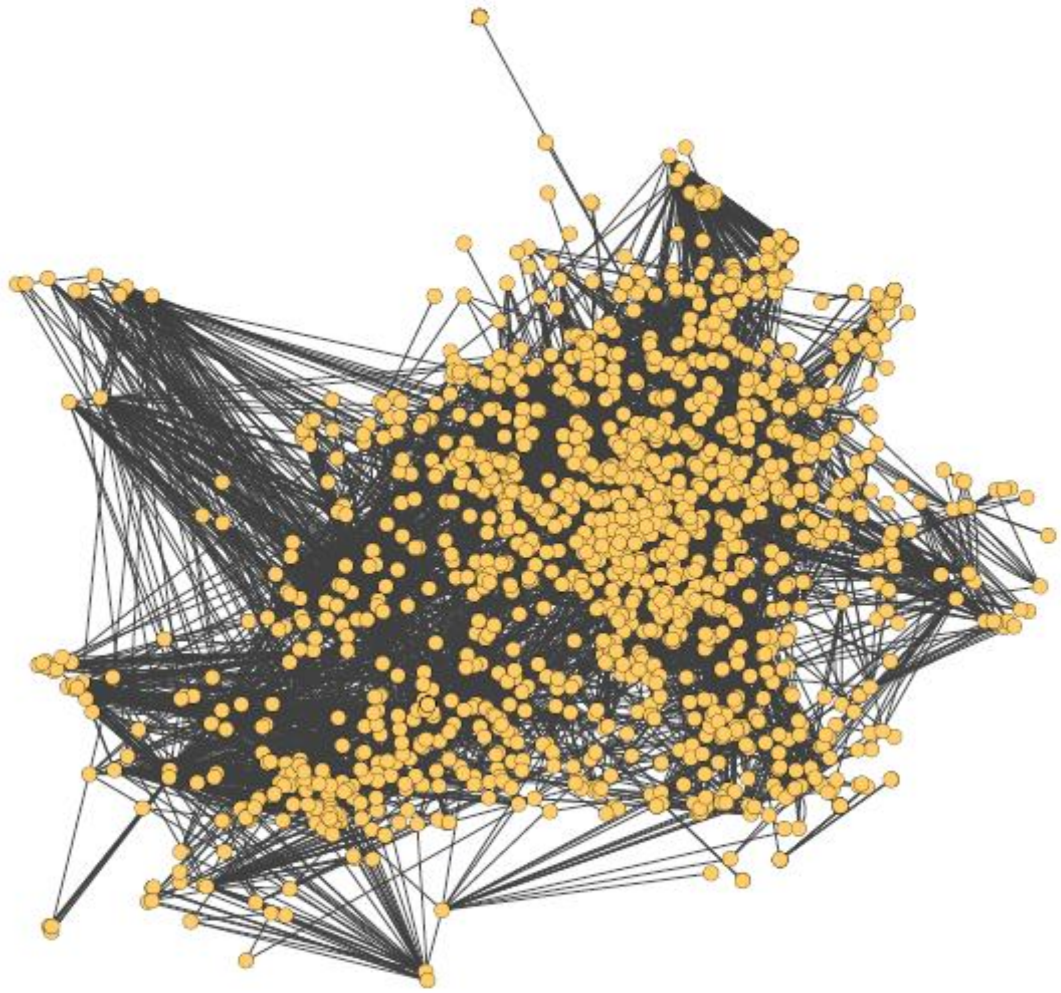

The image was made with the EntOpt Layout program, as described above. The entropy calculations were conducted with the use of the normal colon link weights. The nodes are highlighted with the color yellow.

Figure S14. The EntOpt image of the network with adenoma weights

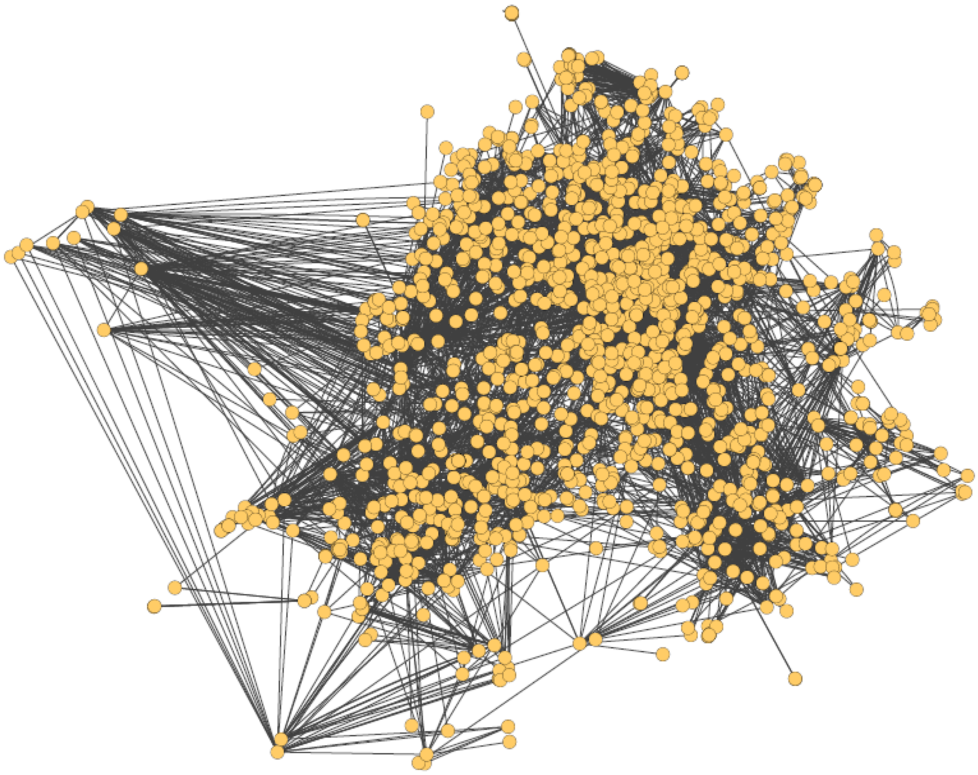

The image was made with the EntOpt Layout program, as described above. The entropy calculations were conducted with the use of the colon adenoma link weights. The nodes are highlighted with the color yellow.

Figure S15. The EntOpt image of the network with carcinoma weights

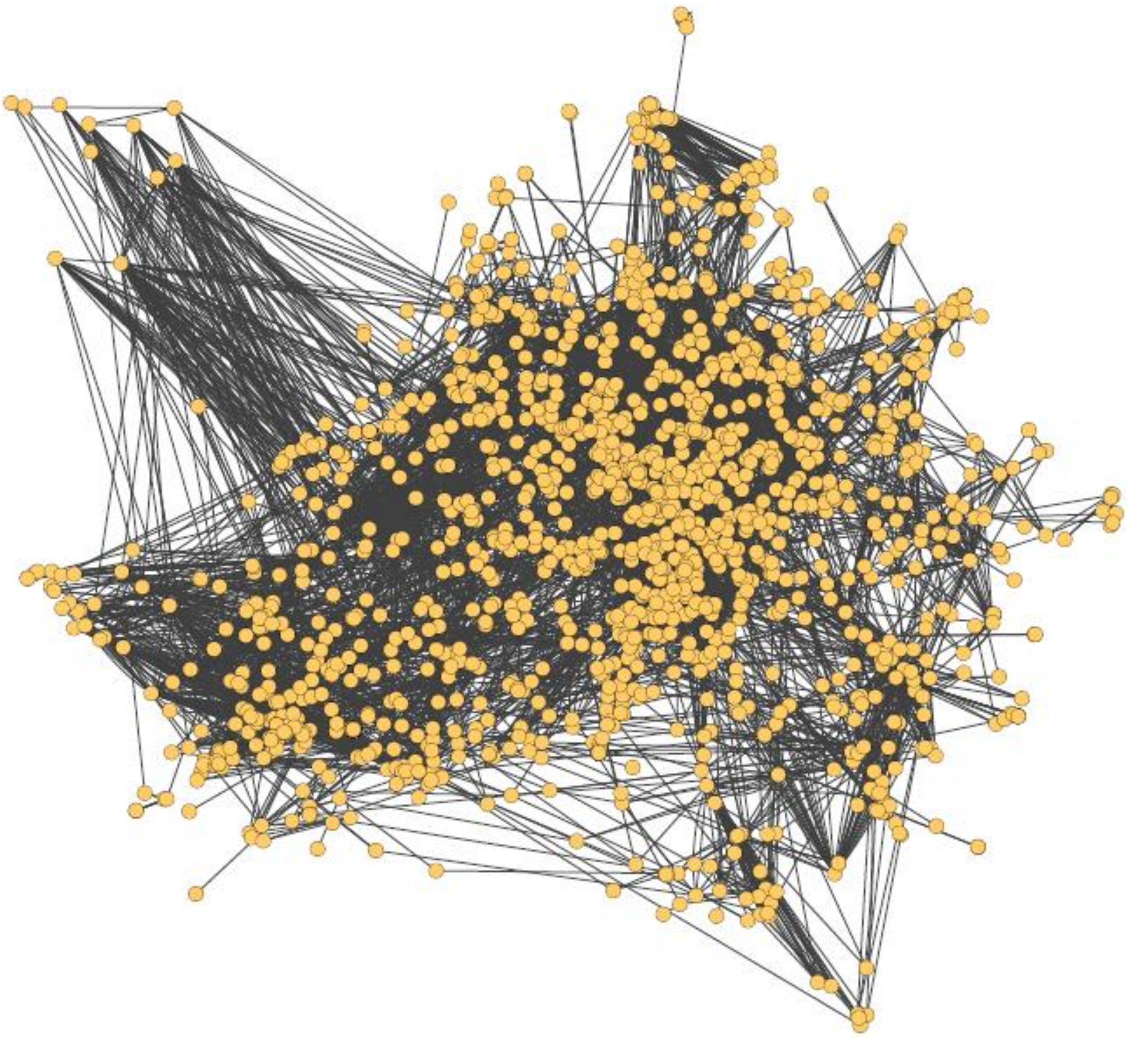

The image was made with the EntOpt Layout program, as described above. The entropy calculations were conducted with the use of the colon carcinoma link weights. The nodes are highlighted with the color yellow.

Figure S16. Change of the ModuLand overlap values based on the different number of modules with logarithmic link weights

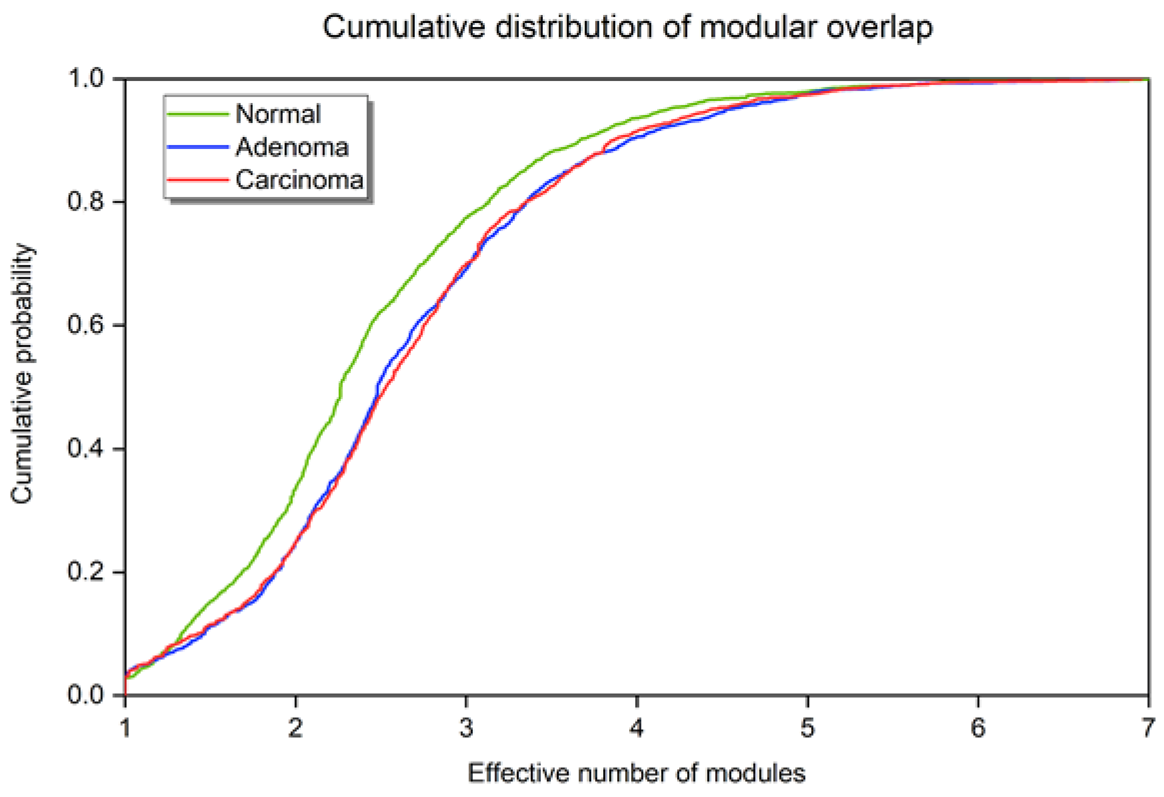

We have investigated into the modular overlap changes, however we found that it strictly relies on the number of modules, which is not a precise entity. Therefore we did not implement this result into the main text.

Figure S17. Change of the ModuLand overlap values based on the different number of modules with non-logarithmic link weights

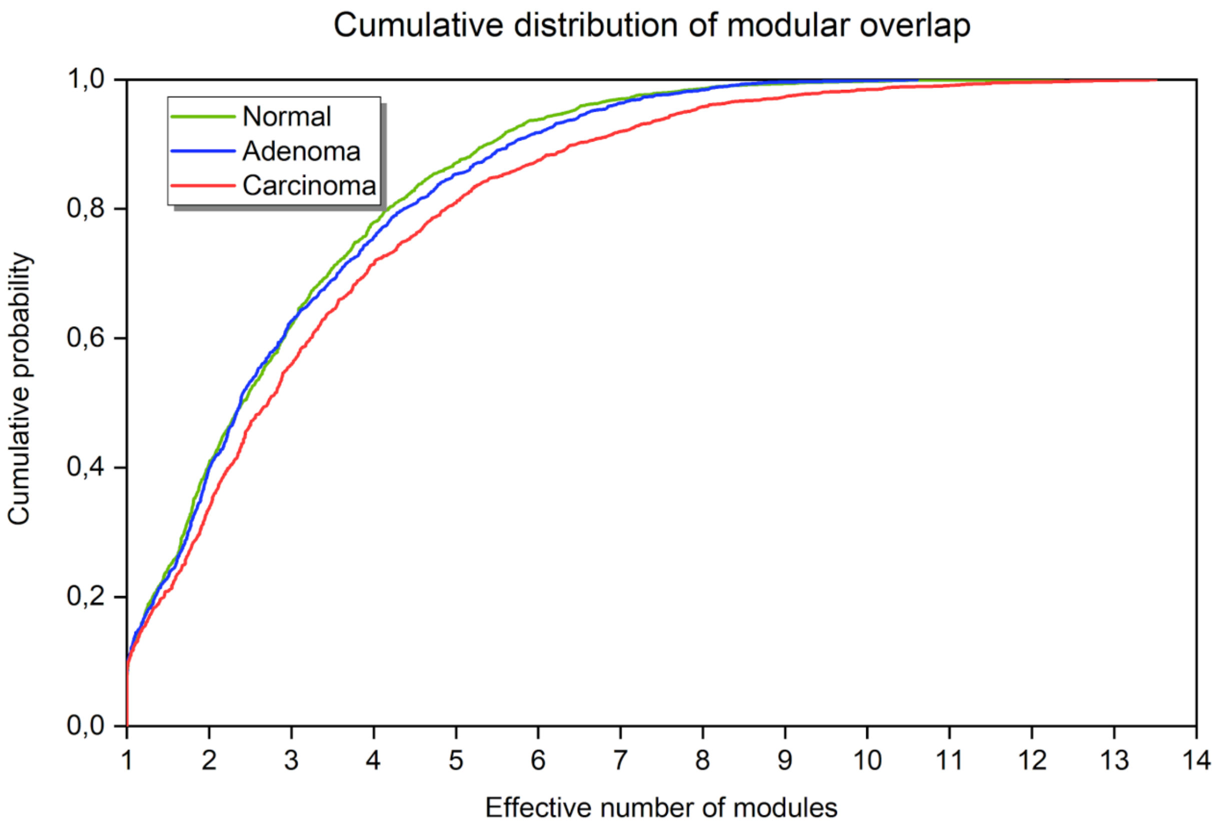

We have investigated into the modular overlap changes, however we found that it strictly relies on the number of modules, which is not a precise entity. Therefore we did not implement this result into the main text.

Figure S18. Cumulative distribution of the effective degree of modules

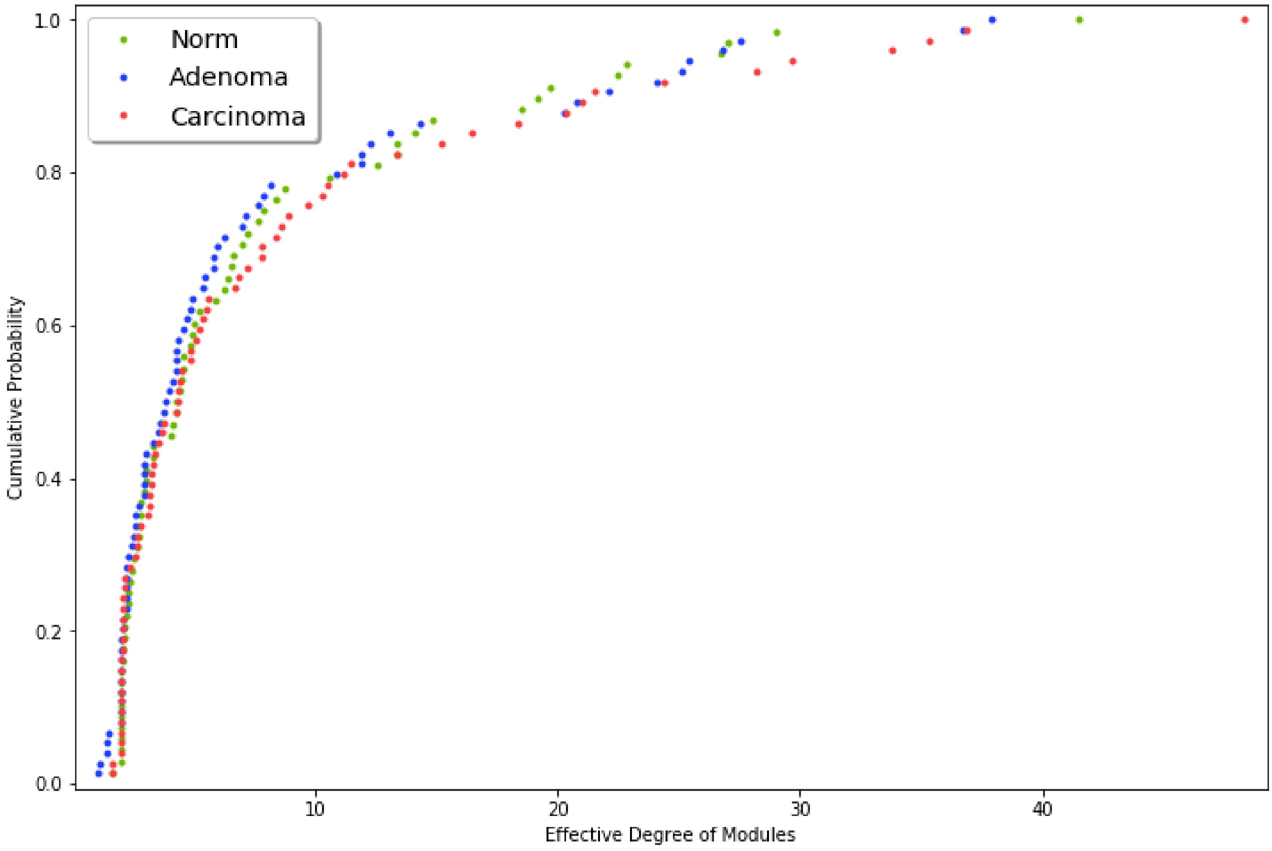

The effective degree of modules were calculated using the ModuLand plugin (see Materials and Methods) from the weighted degree measure of the nodes on the first hierarchical level, each representing a module of the original network. We found that there were no significant changes between the normal, adenoma and carcinoma networks, indicating that the strength of the links between the modules are even. Statistical analysis was performed using Wilcoxon-test. (p_{N-A} = 0.9723, p_{N-C} = 0.1101, p_{A-C}= 0.1314)

Figure S19. Cumulative distribution of the normalized modular overlap

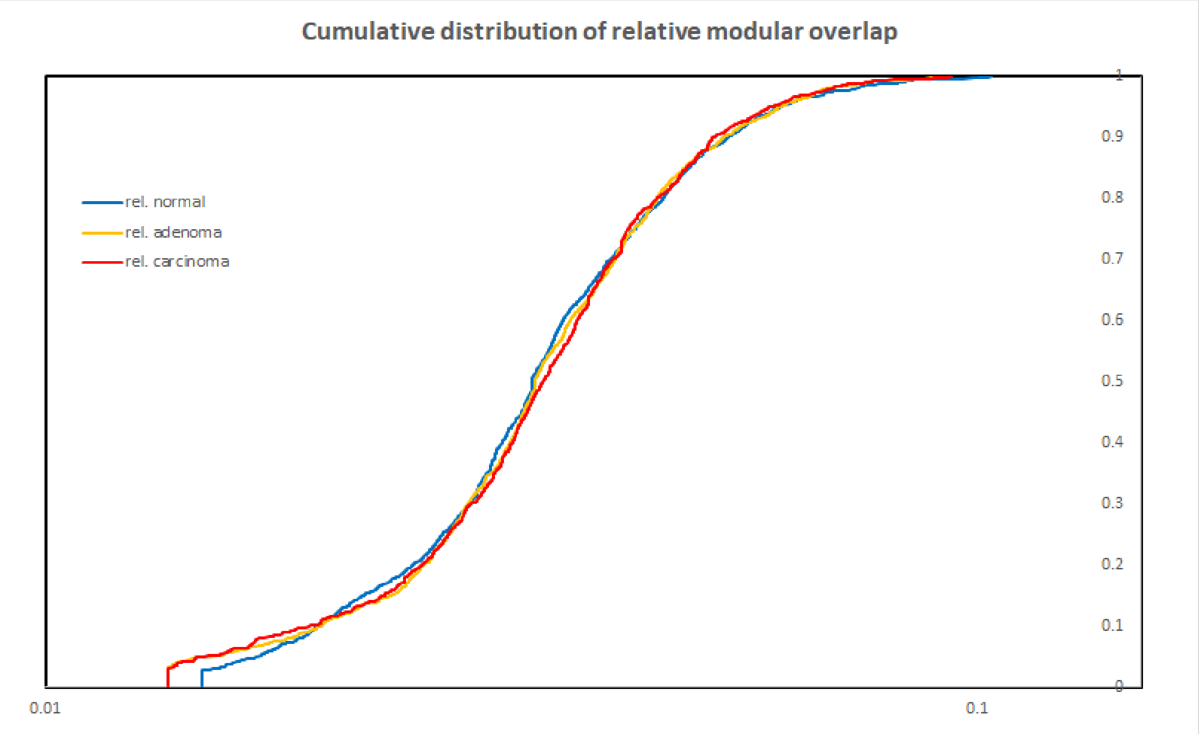

Normalizing the overlap values to the number of modules the difference between normal network versus the adenoma and carcinoma networks almost disappeared, indicating that higher number of modules in the network increases the probability of modular overlap.

Figure S20. EGFR-, VEGFR-signaling and mismatch repair related nodes in the normal network

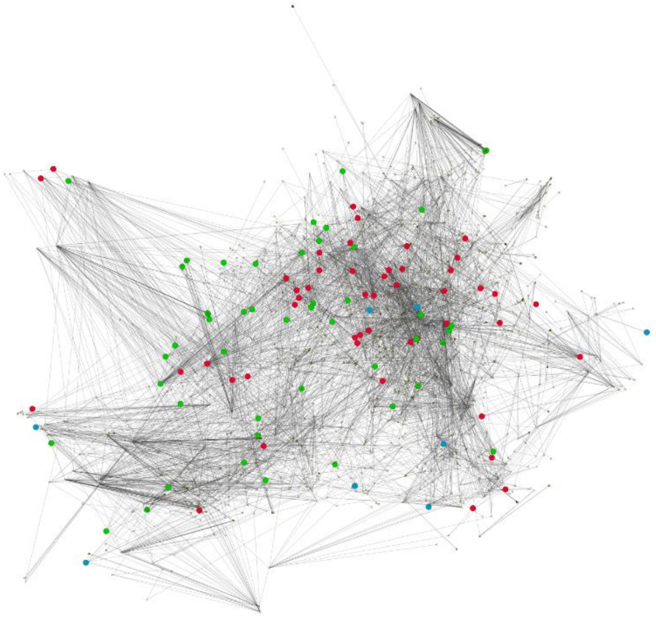

The EntOpt image of the normal network with the coloring of the different pathways. Nodes in the EGFR pathway are green, in the VEGFR pathway are red and in the mismatch repair pathway are blue. As it seems, these nodes do not form different modules, as they are strongly intertwined with the center of the network.

Figure S21. EGFR-, VEGFR-signaling and mismatch repair related nodes in the adenoma network

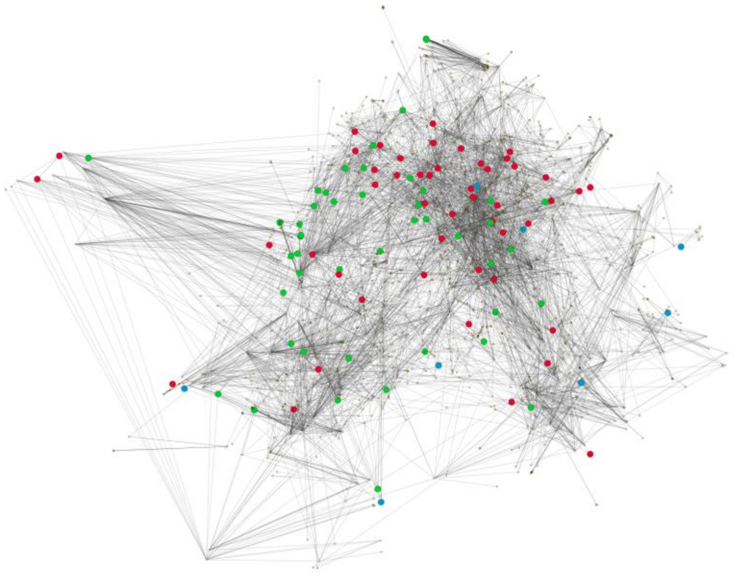

The EntOpt image of the adenoma network with the coloring of the different pathways. Nodes in the EGFR pathway are green, in the VEGFR pathway are red and in the mismatch repair pathway are blue. As it seems, these nodes do not form different modules, as they are strongly intertwined with the center of the network.

Figure S22. EGFR-, VEGFR-signaling and mismatch repair related nodes in the carcinoma network

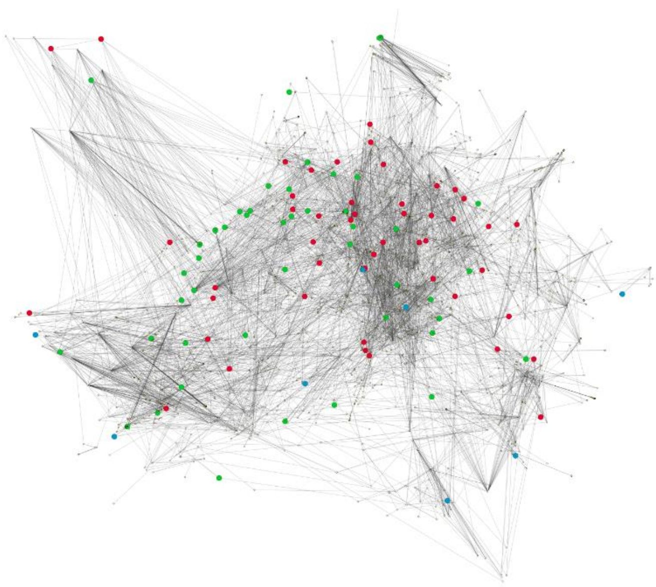

The EntOpt image of the carcinoma network with the coloring of the different pathways. Nodes in the EGFR pathway are green, in the VEGFR pathway are red and in the mismatch repair pathway are blue. As it seems, these nodes do not form different modules, as they are strongly intertwined with the center of the network.

Supporting Tables

Table S1. Number of samples in the dataset series

Supporting Table 1. Number of samples in the dataset series

Series	Normal	Adenoma	Carcinoma	Total
**GSE20916**	44	55	46	145
**GSE33113**	6	0	90	96
**GSE37364**	38	29	27	94
**GSE4183**	8	15	15	38
**GSE8671**	32	32	0	64
**Total**	128	131	178	437

In this research, five GEO data series were processed, which contained normal colon, colon adenoma and adenocarcinoma gene expression data. The distribution of the number of samples is shown by the table above.

Table S2. Network diameter calculations with reciprocal and inverted data, and with additional 5% noise

Table S2.

Network diameters in the normal, adenoma and carcinoma network with negative logarhitmic mapping, and 5% added noise

	**Undirected**[a]		**Directed**[b]		**Mixed graph**[c]	
	**Network diameter**[d]	**Average path length**[e]	**Network diameter**[d]	**Average path length**[e]	**Network diameter**[d]	**Average path length**[e]
**Normal**	34.295	*9.885*	37.346	*12.822*	36.435	*11.399*
**Adenoma**	36.682	*10.573*	40.407	*13.680*	38.876	*12.092*
**Carcinoma**	29.362	*8.122*	32.779	*10.676*	30.552	*9.314*

Network diameters in the normal, adenoma and carcinoma network with reciprocal and inverted data transformation

	**Undirected**[a]		**Directed**[b]		**Mixed graph**[c]	
	**Reciprocal calculation**[f]	**Inverted calculation**[g]	**Reciprocal calculation**[f]	**Inverted calculation**[g]	**Reciprocal calculation**[f]	**Inverted calculation**[g]
**Normal**	44090	*29260*	23739	*21987*	44447	*29697*
**Adenoma**	55649	*38724*	32983	*29065*	57834	*38724*
**Carcinoma**	24523	*20035*	21235	*21235*	31757	*21783*

[a]In the network, every link were assigned as undirected.

[b]The original directivity were preserved in the calculation.

[c]Undirected links were considered as bi-directional links.

[d]Network diameters were calculated with the Dijkstra algorithm.

[e]Average path lengths were calculated with the NetworkX package.

[f]Network diameters were calculated with reciprocal transformation of the data

[g]Network diameters were calculated with inversion of the data

Table S3. Function of the largest network modules nodes

**Table S3.** Estimated functions of the largest modules				
**Name of the module**	**Size (number of nodes)**			**Estimated function**
	Normal	***Adenoma***	***Carcinoma***	
**RAC1**	1000+			signal transduction
**FADD**	38	33	37	death receptor signaling
**GALPHAI**	32	27	33	G protein signaling
**MYD88**	27	28	30	TLR and IL1 signaling (innate immune response)
**CDK1**	25	35	35	cell cycle continuance
**CTNNB1**	22	12	16	WNT signaling pathway
**GAB2**	15	17	9	transcription
**EIF4E**	15	16	17	translation
**BAX**		148	159	mitochondrial apoptosis
**BAD**	128			mitochondrial apoptosis
**CASP3**	43			apoptosis common pathway
**p21**	17			cell cycle control
**CASP7**		13		apoptosis common pathway
**CAMP**			13	protein kinase A-cAMP signaling
**PCNA**			8	cell cycle G1-S transition

Table S4. The largest modules of the networks with additional 5% noise

Table S4. Data with additional 5% noise. Modules of the apoptosis and cell cycle merge together

normal		adenoma		carcinoma	
name	size	name	size	name	size
RAC1	990	RAC1	1025	RAC1	1052
BAD	143	p53	75	BAX	169
FADD	37	BAX	47	CDK1	41
CASP3	35	FADD	36	GALPHAI	34
KIT	35	CASP7	32	MYD88	31
GALPHAI	30	MYD88	30	FADD	29
MYD88	28	GALPHAI	27	EIF4E	17
RB	27	CDK1	24	CTNNB1	15
GAB2	20	GAB2	23	ALK3	10
P21	16	PCNA	19	IFN-yR	10
CGMP	13	EIF4E	16	NASCENTCHAIN	10
ALK3	10	HAT1	11		
HAT1	10	ALK3	10		
IFN-yR	10	CTNNB1	10		
NASCENTCHAIN	10	TRAF2	10		

The apoptosis related modules are highlighted with red.
The cell cycle related modules are highlighted with blue.

Tables S5 and S6. The relevant changes in the strongest and weakest 1% of the links

Table S5. The relevant changes in the strongest 1 % of the links

From (Gene name)	To (Gene name)	Link weight (non-logarithmic)	Module source	Module target	Where?	Link weight change	Relation
HAT1	PTP-SL	1048.722	HAT1	HAT1	Normal	weakening in adenoma, slightly in carcinoma	N > C > A
PTP-SL	HAT1	1048.722	HAT1	HAT1	Normal	weakening in adenoma, slightly in carcinoma	N > C > A
ABP1	PICCOLO	2091.075	ABP1	ABP1	Normal	slightly weakening in adenoma and carcinoma	N > A= C
PAR2	GAQ	2002.359	RAC1	RAC1	Normal	slightly weakening in adenoma and carcinoma	N > A= C
ABP1	DYNAMIN	1595.858	ABP1	RAC1	Normal	slightly weakening in adenoma and carcinoma	N > A= C
cIAP2	CASP7	1324.428	CASP3	CASP3	Normal	slightly weakening in adenoma and carcinoma	N > A= C
FAS	VIL2	1277.447	FADD	RAC1	Normal	slightly weakening in adenoma and carcinoma	N > A= C
cIAP1	CASP7	1184.928	CASP3	CASP3	Normal	slightly weakening in adenoma and carcinoma	N > A= C
SHP1	VAV3	1149.772	GAB2	GAB2	Normal	slightly weakening in adenoma and carcinoma	N > A= C
PKAc	DARPP-32	1134.145	BAD	BAD	Normal	slightly weakening in adenoma and carcinoma	N > A= C
APC	CDH1	1102.694	CTNNB1	RAC1	Normal	slightly weakening in adenoma and carcinoma	N > A= C
MYD88	TLR3	1023.805	MYD88	MYD88	Normal	slightly weakening in adenoma and carcinoma	N > A= C
Calpastatin	VIL2	998.885	RAC1	RAC1	Normal	slightly weakening in adenoma and carcinoma	N > A= C
MAPK13	CEBPA	998.260	RAC1	RAC1	Normal	slightly weakening in adenoma and carcinoma	N > A= C
XIAP	CASP7	957.409	CASP3	CASP3	Normal	slightly weakening in adenoma and carcinoma	N > A= C
CASP7	PROKR1	950.368	CASP3	CASP3	Normal	slightly weakening in adenoma and carcinoma	N > A= C
CASP3	CASP7	922.108	CASP3	CASP3	Normal	slightly weakening in adenoma and carcinoma	N > A= C
CASP3	CASP7	922.108	CASP3	CASP3	Normal	slightly weakening in adenoma and carcinoma	N > A= C
CSNK1D	EPS8	881.357	RAC1	RAC1	Normal	slightly weakening in adenoma and carcinoma	N > A= C
RSK	HAT1	867.247	RAC1	HAT1	Normal	slightly weakening in adenoma and carcinoma	N > A= C
IFN-yR	TIP1	863.428	IFN-yR	IFN-yR	Normal	slightly weakening in adenoma and carcinoma	N > A= C
MEK6	MAPK13	853.835	RAC1	RAC1	Normal	slightly weakening in adenoma and carcinoma	N > A= C
CLCA1	ITGB4	5597.775	RAC1	RAC1	Normal	slightly weakening in carcinoma	N = A > C
BETAARRESTIN	PAR2	1840.333	RAC1	RAC1	Normal	slightly weakening in carcinoma	N = A > C
SHC	CEACAM1	1813.733	RAC1	RAC1	Normal	slightly weakening in carcinoma	N = A > C
CDH1	SMAD3	1715.162	RAC1	RAC1	Normal	slightly weakening in carcinoma	N = A > C
PAR2	GBETAGAMMA	1634.350	RAC1	RAC1	Normal	slightly weakening in carcinoma	N = A > C
EPS8	TCF4	1459.934	RAC1	GAB2	Normal	slightly weakening in carcinoma	N = A > C
ERT	EF1A	1103.203	RAC1	RAC1	Normal	slightly weakening in carcinoma	N = A > C
DARPP-32	PPP1CC	1029.393	BAD	BAD	Normal	slightly weakening in carcinoma	N = A > C
CD151	ITGA6	1788.993	RAC1	ITGA6	Adenoma	slightly weakening in normal and carcinoma	A > N = C

DARPP-32	PP1	1584.007	DARP P-32	DAR PP-32	Adenoma	slightly weakening in normal and carcinoma	A > N = C
BPAG2	ITGA6	1311.550	RAC1	ITGA 6	Adenoma	slightly weakening in normal and carcinoma	A > N = C
PRKCD	ITGA6	1205.886	RAC1	ITGA 6	Adenoma	slightly weakening in normal and carcinoma	A > N = C
HES1	ID1	1109.190	RAC1	RAC1	Adenoma	slightly weakening in normal and carcinoma	A > N = C
CASP6	LMNB2	1030.340	CASP7	CAS P7	Adenoma	slightly weakening in normal and carcinoma	A > N = C
CCNB1	CDK1	3663.603	CDK1	CDK1	Adenoma	slightly weakening in normal	A = C > N
CCNB1	CDK1	3663.603	CDK1	CDK1	Adenoma	slightly weakening in normal	A = C > N
CDK1	CCNB1	3663.603	CDK1	CDK1	Adenoma	slightly weakening in normal	A = C > N
LAMR1	ITGA6	2558.331	ITGA6	ITGA 6	Adenoma	slightly weakening in normal	A = C > N
p53	BIK	2347.750	BAX	BAX	Adenoma	slightly weakening in normal	A = C > N
CCNA2	CDK1	1734.955	CDK1	CDK1	Adenoma	slightly weakening in normal	A = C > N
CCNA2	CDK1	1734.955	CDK1	CDK1	Adenoma	slightly weakening in normal	A = C > N
HMG1	TopoII	1603.390	HMG1	HMG 1	Adenoma	slightly weakening in normal	A = C > N
CDK4	PCNA	1603.026	CDK4	CDK4	Adenoma	slightly weakening in normal	A = C > N
PCNA	CDK4	1603.026	CDK4	CDK4	Adenoma	slightly weakening in normal	A = C > N
CD44	EPS8	1320.956	RAC1	RAC1	Adenoma	slightly weakening in normal	A = C > N
BLNK	LYN	1308.223	SYK	SYK	Adenoma	slightly weakening in normal	A = C > N
RSK2	HAT1	1194.485	RAC1	HAT1	Adenoma	slightly weakening in normal	A = C > N
EPS8	RUVBL1	1151.805	RAC1	RAC1	Adenoma	slightly weakening in normal	A = C > N
HAT1	UXT	1065.008	HAT1	HAT1	Adenoma	slightly weakening in normal	A = C > N
UXT	HAT1	1065.008	HAT1	HAT1	Adenoma	slightly weakening in normal	A = C > N
SYNDECAN	SYNTENIN	1063.515	SYNTE NIN	SYNT ENIN	Adenoma	slightly weakening in normal	A = C > N
LMNB1	LMNB2	1016.402	CASP7	CAS P7	Adenoma	slightly weakening in normal	A = C > N
CLCA1	ITGB4	6477.399	RAC1	RAC1	Adenoma	slightly weakening in carcinoma	A = N > C
CDH1	SMAD3	1287.473	RAC1	RAC1	Adenoma	slightly weakening in carcinoma	A = N > C
DARPP-32	PPP1CC	1043.032	DARP P-32	BAX	Adenoma	slightly weakening in carcinoma	A = N > C
ERT	EF1A	1005.412	RAC1	RAC1	Adenoma	slightly weakening in carcinoma	A = N > C
EPS8	TCF4	999.437	RAC1	GAB2	Adenoma	slightly weakening in carcinoma	A = N > C
PAR2	GBETAGAM MA	973.656	RAC1	RAC1	Adenoma	slightly weakening in carcinoma	A = N > C
CCNA2	CDK4	1019.792	CDK1	PCN A	Carcinoma	weakening in normal, slightly in adenoma	C > A > N
HIF1A	Noxa	1107.099	BAX	BAX	Carcinoma	weakening in normal, slightly in adenoma	C > A > N
CDK1	PLK1	1343.745	CDK1	CDK1	Carcinoma	weakening in normal, slightly in adenoma	C > A > N
IL1A	IL8	1347.597	FADD	RAC1	Carcinoma	weakening in normal and adenoma	C >> A = N
RELA	IL8	2210.242	RAC1	RAC1	Carcinoma	weakening in normal, slightly in adenoma	C > A > N
HES1	PLSCR1	971.908	RAC1	RAC1	Carcinoma	slightly weakening in normal and adenoma	C > A = N
STAT1	PLSCR1	1172.367	RAC1	RAC1	Carcinoma	slightly weakening in normal and adenoma	C > A = N
Importin	RAN	1226.940	RAC1	RAC1	Carcinoma	slightly weakening in normal and adenoma	C > A = N
Importin	RAN	1226.940	RAC1	RAC1	Carcinoma	slightly weakening in normal and adenoma	C > A = N
PLSCR1	PTPN12	1229.324	RAC1	RAC1	Carcinoma	slightly weakening in normal and adenoma	C > A = N
PTPN12	PLSCR1	1229.324	RAC1	RAC1	Carcinoma	slightly weakening in normal and adenoma	C > A = N

CCND1	PCNA	1283.714	PCNA	PCN A	Carcinoma	slightly weakening in normal and adenoma	C > A = N
HAT1	UXT	949.671	HAT1	HAT1	Carcinoma	slightly weakening in normal	C = A > N
UXT	HAT1	949.671	HAT1	HAT1	Carcinoma	slightly weakening in normal	C = A > N
BLNK	LYN	967.056	LYN	RAC1	Carcinoma	slightly weakening in normal	C = A > N
p53	BIK	1022.720	BAX	BAX	Carcinoma	slightly weakening in normal	C = A > N
SYNDECAN	SYNTENIN	1060.180	SYNTE NIN	SYNT ENIN	Carcinoma	slightly weakening in normal	C = A > N
RSK2	HAT1	1068.930	RAC1	HAT1	Carcinoma	slightly weakening in normal	C = A > N
LMNB1	LMNB2	1146.680	BAX	BAX	Carcinoma	slightly weakening in normal	C = A > N
CD44	EPS8	1196.907	RAC1	RAC1	Carcinoma	slightly weakening in normal	C = A > N
EPS8	RUVBL1	1268.265	RAC1	RAC1	Carcinoma	slightly weakening in normal	C = A > N
LAMR1	ITGA6	1421.976	ITGA6	ITGA 6	Carcinoma	slightly weakening in normal	C = A > N
HMG1	TopoII	1803.851	HMG1	HMG 1	Carcinoma	slightly weakening in normal	C = A > N
CDK4	PCNA	1842.590	PCNA	PCN A	Carcinoma	slightly weakening in normal	C = A > N
PCNA	CDK4	1842.590	PCNA	PCN A	Carcinoma	slightly weakening in normal	C = A > N
CCNA2	CDK1	2834.158	CDK1	CDK1	Carcinoma	slightly weakening in normal	C = A > N
CCNA2	CDK1	2834.158	CDK1	CDK1	Carcinoma	slightly weakening in normal	C = A > N
CCNB1	CDK1	5345.275	CDK1	CDK1	Carcinoma	slightly weakening in normal	C = A > N
CCNB1	CDK1	5345.275	CDK1	CDK1	Carcinoma	slightly weakening in normal	C = A > N
CDK1	CCNB1	5345.275	CDK1	CDK1	Carcinoma	slightly weakening in normal	C = A > N

Supporting Table 6. The relevant changes in the weakest 1% of the links

From (Gene name)	To (Gene name)	Link weight (non-logarithmic)	Module source	Module target	Where?	Link weight change	Relation
AR	WNT2	5.999	RAC1	RAC1	Normal	strengthening in carcinoma, slightly in adenoma	C > A > N
EGR2	BNIP3	6.406	BAD	BAD	Normal	strengthening in carcinoma, slightly in adenoma	C > A > N
RELN	APOER2	6.473	VLDLR	VLDLR	Normal	strengthening in carcinoma, slightly in adenoma	C > A > N
AGTR2	AGT	6.868	RAC1	RAC1	Normal	strengthening in adenoma and carcinoma	C = A >> N
SYNAPTOTAGMIN	LTYPECA	4.339	RAC1	RAC1	Normal	slightly strengthening in carcinoma	C > A = N
CAMK2	IL1A	5.838	RAC1	FADD	Normal	slightly strengthening in adenoma and carcinoma	C = A > N
BMP-7	BMPR1	5.982	ALK3	ALK3	Normal	slightly strengthening in adenoma and carcinoma	C = A > N
WIF1	WNT	6.063	RAC1	RAC1	Normal	slightly strengthening in adenoma and carcinoma	C = A > N
AR	RAC3	6.781	RAC1	RAC1	Normal	slightly strengthening in adenoma and carcinoma	C = A > N
WNT	AR	6.805	RAC1	RAC1	Normal	slightly strengthening in adenoma and carcinoma	C = A > N
AMPHIPHYSIN	DYNAMIN	6.835	RAC1	RAC1	Normal	slightly strengthening in adenoma and carcinoma	C = A > N
cAMPGEFII	RIM	6.873	RAC1	RAC1	Normal	slightly strengthening in adenoma and carcinoma	C = A > N
cAMPGEFII	RIM	6.873	RAC1	RAC1	Normal	slightly strengthening in adenoma and carcinoma	C = A > N
AR	SLC25A4	6.889	RAC1	RAC1	Normal	slightly strengthening in adenoma and carcinoma	C = A > N
ANKYRIN	NRCAM	6.894	RAC1	RAC1	Normal	slightly strengthening in adenoma and carcinoma	C = A > N
NMDAR	RACK	6.909	RAC1	RAC1	Normal	slightly strengthening in adenoma and carcinoma	C = A > N
JNK3	RNPK	6.923	RAC1	RAC1	Normal	slightly strengthening in adenoma and carcinoma	C = A > N
GALPHAZ	AC5	6.955	RAC1	RAC1	Normal	slightly strengthening in adenoma and carcinoma	C = A > N
BMP-10	BMPR1	5.445	ALK3	ALK3	Normal	slightly strengthening in adenoma	A > C = N
AR	SRY	6.087	RAC1	RAC1	Normal	slightly strengthening in adenoma	A > C = N
PKA	TAU	6.441	RAC1	RAC1	Normal	slightly strengthening in adenoma	A > C = N
AMPAR	HOMER	6.756	RAC1	RAC1	Normal	slightly strengthening in adenoma	A > C = N
SMAD4	AR	6.815	RAC1	RAC1	Normal	slightly strenghtening in carcinoma	C > A = N
RELN	VLDLR	6.153	DAB1	DAB1	Adenoma	strengthening in normal, slightly in carcinoma	N > C > A
SMAD4	FORKHEAD	5.788	RAC1	RAC1	Adenoma	strengthening in carcinoma, slightly in normal	C > N > A
CryAB	CASP3	3.874	BAX	BAX	Adenoma	slightly strengthening in normal and carcinoma	C = N > A
AR	FORKHEAD	4.418	RAC1	RAC1	Adenoma	slightly strengthening in normal and carcinoma	C = N > A
ABI2	WAVE3	4.562	RAC1	RAC1	Adenoma	slightly strengthening in normal and carcinoma	C = N > A
MAP1B	TUBULIN	4.851	RAC1	RAC1	Adenoma	slightly strengthening in normal and carcinoma	C = N > A
NIK	WAVE1	4.926	RAC1	RAC1	Adenoma	slightly strengthening in normal and carcinoma	C = N > A
PCAF	AR	5.327	RAC1	RAC1	Adenoma	slightly strengthening in normal and carcinoma	C = N > A
JIP	TIAM1	5.330	RAC1	RAC1	Adenoma	slightly strengthening in normal and carcinoma	C = N > A
NMDAR	YOTIAO	5.336	RAC1	RAC1	Adenoma	slightly strengthening in normal and carcinoma	C = N > A
APAF1	WAVE3	5.567	BAX	RAC1	Adenoma	slightly strengthening in normal and carcinoma	C = N > A
WAVE3	APAF1	5.567	RAC1	BAX	Adenoma	slightly strengthening in normal and carcinoma	C = N > A

FYN	NMDAR	5.901	RAC1	RAC1	Adenoma	slightly strengthening in normal and carcinoma	C = N > A
SMAD4	ZFHX1B	5.920	RAC1	RAC1	Adenoma	slightly strengthening in normal and carcinoma	C = N > A
RACK	FYN	6.016	RAC1	RAC1	Adenoma	slightly strengthening in normal and carcinoma	C = N > A
FYN	AMPAR	6.069	RAC1	RAC1	Adenoma	slightly strengthening in normal and carcinoma	C = N > A
AR	GP130	5.175	RAC1	RAC1	Adenoma	slightly strengthening in normal	N > C = A
TRKB	FYN	5.650	RAC1	RAC1	Adenoma	slightly strengthening in normal	N > C = A
PKCA	SNAP25	5.665	RAC1	SYNTAXIN	Adenoma	slightly strengthening in normal	N > C = A
CAMK2A	TIAM1	5.755	RAC1	RAC1	Adenoma	slightly strengthening in normal	N > C = A
ANKYRIN	FASCIN	5.849	RAC1	RAC1	Adenoma	slightly strengthening in normal	N > C = A
SMAD4	AR	5.004	RAC1	RAC1	Adenoma	slightly strengthening in carcinoma	N > C = A
SYNAPTOTAGMIN	LTYPECA	5.826	RAC1	RAC1	Adenoma	slightly strengthening in carcinoma	N > C = A
NTF5	TRKB	5.180	RAC1	RAC1	Carcinoma	slightly strengthening in normal and adenoma	N = A > C
NEUROLIGIN	PSD95	5.181	RAC1	RAC1	Carcinoma	slightly strengthening in normal and adenoma	N = A > C
M2R	GALPHAO	5.451	GALPHAI	GALPHAI	Carcinoma	slightly strengthening in normal and adenoma	N = A > C
CB1R	GALPHAO	5.739	GALPHAI	GALPHAI	Carcinoma	slightly strengthening in normal and adenoma	N = A > C
M4R	EEF1A2	5.775	GALPHAI	GALPHAI	Carcinoma	slightly strengthening in normal and adenoma	N = A > C
AR	CDK9	5.803	RAC1	RAC1	Carcinoma	slightly strengthening in normal and adenoma	N = A > C
NOS1	PSD95	5.852	RAC1	RAC1	Carcinoma	slightly strengthening in normal and adenoma	N = A > C
SSTR2	GALPHAO	6.027	GALPHAI	GALPHAI	Carcinoma	slightly strengthening in normal and adenoma	N = A > C
MOPR	GALPHAO	6.104	GALPHAI	GALPHAI	Carcinoma	slightly strengthening in normal and adenoma	N = A > C
KOPR	GALPHAO	6.125	GALPHAI	GALPHAI	Carcinoma	slightly strengthening in normal and adenoma	N = A > C
BDNF	TRKB	6.156	RAC1	RAC1	Carcinoma	slightly strengthening in normal and adenoma	N = A > C
NOS1	PSD93	6.182	RAC1	RAC1	Carcinoma	slightly strengthening in normal and adenoma	N = A > C
KV12	PSD95	6.208	RAC1	RAC1	Carcinoma	slightly strengthening in normal and adenoma	N = A > C
CHAPSYN 110	PSD95	6.309	RAC1	RAC1	Carcinoma	slightly strengthening in normal and adenoma	N = A > C
ANKYRIN	FASCIN	5.103	RAC1	RAC1	Carcinoma	slightly strengthening in normal	N > A = C
PKCA	SNAP25	6.092	RAC1	RAC1	Carcinoma	slightly strengthening in normal	N > A = C
CAMK2A	TIAM1	6.229	RAC1	RAC1	Carcinoma	slightly strengthening in normal	N > A = C
TRKB	FYN	6.307	RAC1	RAC1	Carcinoma	slightly strengthening in normal	N > A = C
BMP-10	BMPR1	5.084	ALK3	ALK3	Carcinoma	slightly strengthening in adenoma	A > C = N
PKA	TAU	5.579	RAC1	RAC1	Carcinoma	slightly strengthening in adenoma	A > C = N
AMPAR	HOMER	5.656	RAC1	RAC1	Carcinoma	slightly strengthening in adenoma	A > C = N
AR	SRY	5.837	RAC1	RAC1	Carcinoma	slightly strengthening in adenoma	A > C = N

Table S7. The relevant changes in the strongest and weakest 1% of the links with additional 5% noise

Table S7. Top 1% of linkweights with additional 5% of noise

From (Gene name)	To (Gene name)	Noisy_linkweight	Module_source	Module_target
Normal				
CASP6	CASP7	3.400	**CASP3**	**CASP3**
CASP6	LMNB1	3.316	**CASP3**	**CASP3**
cIAP2	CASP7	2.966	**CASP3**	**CASP3**
PKAc	DARPP-32	3.207	**BAD**	**BAD**
cIAP1	CASP7	2.920	**CASP3**	**CASP3**
DARPP-32	PPP1CC	3.163	**BAD**	**BAD**
TFF1	APAF1	2.901	**RAC1**	**BAD**
CASP3	CASP7	3.113	**CASP3**	**CASP3**
XIAP	CASP7	2.832	**CASP3**	**CASP3**
CASP7	PROKR1	2.829	**CASP3**	**CASP3**
CASP3	CASP7	2.816	**CASP3**	**CASP3**
Adenoma				
**From (Gene name)**	**To (Gene name)**	**Noisy_linkweight**	**Module_source**	**Module_target**
CCNB1	CDK1	3.742	**CDK1**	**CDK1**
CDK1	CCNB1	3.386	**CDK1**	**CDK1**
CCNB1	CDK1	3.386	**CDK1**	**CDK1**
CASP6	CASP7	3.559	**CASP7**	**CASP7**
p53	BIK	3.539	**p53**	**p53**
CASP6	LMNB1	3.491	**CASP7**	**CASP7**
CCNA2	CDK1	3.401	**CDK1**	**CDK1**
CCNA2	CDK1	3.077	**CDK1**	**CDK1**
PCNA	CDK4	3.045	**PCNA**	**PCNA**
CDK4	PCNA	3.045	**PCNA**	**PCNA**
CASP6	LMNB2	3.164	**CASP7**	**CASP7**
p53	FAS	3.137	**p53**	**FADD**
CCND1	PCNA	3.115	**PCNA**	**PCNA**
Carcinoma				
**From (Gene name)**	**To (Gene name)**	**Noisy_linkweight**	**Module_source**	**Module_target**
CDK1	CCNB1	3.914	**CDK1**	**CDK1**
CCNB1	CDK1	3.542	**CDK1**	**CDK1**
CCNB1	CDK1	3.542	**CDK1**	**CDK1**
CCNA2	CDK1	3.625	**CDK1**	**CDK1**
CCNA2	CDK1	3.625	**CDK1**	**CDK1**
CDK4	PCNA	3.429	**PCNA**	**PCNA**
PCNA	CDK4	3.429	**PCNA**	**PCNA**
CASP6	LMNB1	3.025	**BAX**	**BAX**
CCND1	PCNA	3.264	**PCNA**	**PCNA**

CASP6	CASP7	3.261	**BAX**	**CASP7**
CDK1	PLK1	2.972	**CDK1**	**CDK1**
LMNB1	LMNB2	2.906	**BAX**	**BAX**
CCNA2	CDK4	3.159	**CDK1**	**PCNA**
HIF1A	Noxa	2.892	**BAX**	**BAX**
GADD45	CDK1	3.120	**CDK1**	**CDK1**
p53	BIK	2.859	**BAX**	**BAX**

Modules highlighted with blue belong to the cell cycle regulation process,
Modules highlighted with red belong to the apoptosis regulation process.

We have listed here only the link weights belonging to the apoptosis or cell cycle related modules. We have found that the number of cell cycle related links is increasing in the adenoma network and even more in the carcinoma network.

Table S8. Representation of the apoptosis and cell cycle related modules among strongest and weakest 10% of the abundances, weighted degrees and the link weights

Table S8. Ratio of cell cycle and apoptosis related modules among the top 10% of mRNA abundances, node degrees and link weights

mRNA abundances

Modules	top 10% normal Count	%	top 10% adenoma Count	%	top 10% carcinoma Count	%	bottom 10% normal Count	%	bottom 10% adenoma Count	%	bottom 10% carcinoma Count	%
**BAD**	14	*8.75%*	0	*0.00%*	0	*0.00%*	9	*5.63%*	0	*0.00%*	0	*0.00%*
**CASP3**	5	*3.13%*	0	*0.00%*	0	*0.00%*	3	*1.88%*	0	*0.00%*	0	*0.00%*
**CDK1**	4	*2.50%*	5	*3.13%*	4	*2.50%*	1	*0.63%*	1	*0.63%*	1	*0.63%*
**CDK7**	1	*0.63%*	1	*0.63%*	1	*0.63%*	0	*0.00%*	0	*0.00%*	0	*0.00%*
**FADD**	3	*1.88%*	2	*1.25%*	4	*2.50%*	3	*1.88%*	0	*0.00%*	1	*0.63%*
**P21**	1	*0.63%*	0	*0.00%*	0	*0.00%*	0	*0.00%*	0	*0.00%*	0	*0.00%*
**BAX**	0	*0.00%*	16	*10.00%*	22	*13.75%*	0	*0.00%*	12	*7.50%*	10	*6.25%*
**CASP7**	0	*0.00%*	5	*3.13%*	1	*0.63%*	0	*0.00%*	1	*0.63%*	0	*0.00%*
**CDK4**	0	*0.00%*	3	*1.88%*	0	*0.00%*	0	*0.00%*	0	*0.00%*	0	*0.00%*
**CDK9**	0	*0.00%*	0	*0.00%*	0	*0.00%*	0	*0.00%*	0	*0.00%*	0	*0.00%*
**CAMP**	0	*0.00%*	0	*0.00%*	0	*0.00%*	0	*0.00%*	0	*0.00%*	0	*0.00%*
**PCNA**	0	*0.00%*	0	*0.00%*	2	*1.25%*	0	*0.00%*	0	*0.00%*	0	*0.00%*

Node degrees

Modules	top 10% normal Count	%	top 10% adenoma Count	%	top 10% carcinoma Count	%	bottom 10% normal Count	%	bottom 10% adenoma Count	%	bottom 10% carcinoma Count	%
**BAD**	17	*10.63%*	0	*0.00%*	0	*0.00%*	12	*7.50%*	0	*0.00%*	0	*0.00%*
**CASP3**	6	*3.75%*	0	*0.00%*	0	*0.00%*	3	*1.88%*	0	*0.00%*	0	*0.00%*
**FADD**	6	*3.75%*	5	*3.13%*	6	*3.75%*	2	*1.25%*	2	*1.25%*	3	*1.88%*
**CDK1**	3	*1.88%*	5	*3.13%*	5	*3.13%*	4	*2.50%*	6	*3.75%*	5	*3.13%*
**P21**	1	*0.63%*	0	*0.00%*	0	*0.00%*	2	*1.25%*	0	*0.00%*	0	*0.00%*
**BAX**	0	*0.00%*	19	*11.88%*	23	*14.38%*	0	*0.00%*	15	*9.38%*	12	*7.50%*
**CASP7**	0	*0.00%*	4	*2.50%*	1	*0.63%*	0	*0.00%*	0	*0.00%*	0	*0.00%*

Link weights

Modules	top 10% normal Count	%	top 10% adenoma Count	%	top 10% carcinoma Count	%	bottom 10% normal Count	%	bottom 10% adenoma Count	%	bottom 10% carcinoma Count	%
**BAD**	57	*11.40%*	0	*0.00%*	0	*0.00%*	23	*4.60%*	0	*0.00%*	0	*0.00%*
**CASP3**	34	*6.80%*	0	*0.00%*	0	*0.00%*	0	*0.00%*	0	*0.00%*	0	*0.00%*
**CDK1**	9	*1.80%*	23	*4.60%*	27	*5.40%*	1	*0.20%*	1	*0.20%*	2	*0.40%*
**CDK7**	1	*0.20%*	2	*0.40%*	2	*0.40%*	0	*0.00%*	0	*0.00%*	0	*0.00%*
**FADD**	19	*3.80%*	16	*3.20%*	19	*3.80%*	4	*0.80%*	1	*0.20%*	0	*0.00%*
**P21**	8	*1.60%*	0	*0.00%*	0	*0.00%*	1	*0.20%*	0	*0.00%*	0	*0.00%*

**BAX**	0	*0.00%*	83	*16.60%*	90	*18.00%*	0	*0.00%*	20	*4.00%*	15	*3.00%*
**CASP7**	0	*0.00%*	24	*4.80%*	6	*1.20%*	0	*0.00%*	0	*0.00%*	0	*0.00%*
**CDK4**	0	*0.00%*	7	*1.40%*	0	*0.00%*	0	*0.00%*	0	*0.00%*	0	*0.00%*
**CDK9**	0	*0.00%*	0	*0.00%*	0	*0.00%*	0	*0.00%*	0	*0.00%*	0	*0.00%*
**PCNA**	0	*0.00%*	0	*0.00%*	7	*1.40%*	0	*0.00%*	0	*0.00%*	0	*0.00%*

Table S9. The nodes in the targeted and immunotherapy related pathways

**Table S9.** Node in targeted and immunotherapy pathways				
**EGFR signaling**				
**UniProt**	**Name**	**Normal abundance**	**Adenoma abundance**	**Carcinoma abundance**
Q99962	ENDOPHILIN	2.087	1.940	1.607
P01019	AGT	2.397	5.715	6.842
O00291	HIP1	2.831	2.810	4.094
Q14155	P50	3.012	2.924	3.385
P22681	CBL	3.320	3.478	3.375
P30542	A1R	3.360	3.189	2.857
P48023	FASLG	3.715	3.322	3.245
Q05397	FAK	3.965	4.972	5.921
P01133	EGF	4.332	6.502	3.072
O14964	HGS	4.358	4.371	4.951
Q969H0	FBW7	4.825	4.618	5.306
Q99075	HBEGF	4.845	4.958	7.201
P19174	PLCy	4.896	4.982	5.679
P56199	ITGA1	4.942	4.814	10.522
O15530	PDPK1	5.046	4.860	4.399
P42336	PI3K	5.087	4.688	5.842
P18545	PDE6G	5.114	4.935	4.537
Q9P212	PLCE1	5.202	4.869	4.627
P12931	SRC	5.240	5.415	5.119
P15056	bRAF	5.279	5.998	6.407
P27986	P13K	5.390	5.584	4.955
P16591	FER	5.461	5.426	5.892
P78536	ADAM17	5.474	6.019	7.266
P56945	P130Cas	5.798	5.010	4.973
O14944	ER	5.956	4.390	19.968
P01137	TGFB	6.250	5.380	7.047
Q06124	SHP2	6.286	7.684	8.457
Q99704	DOK	6.343	7.151	7.068
P42566	EPS15	6.980	6.085	6.803
Q05209	PTPN12	7.141	9.643	23.956
O75582	MSK1	7.264	6.118	5.671
Q14289	PTK	7.421	6.059	6.103
Q13191	CBLB	7.824	8.672	8.806
O43609	SPRY	7.826	7.182	9.386
Q14451	Grb7	7.969	10.744	11.390
Q9UJM3	MIG6	8.146	8.693	19.414
P00533	EGFR	8.746	8.065	5.623

O75159	SOCS5	8.871	8.789	10.222
Q13480	GAB1	9.354	6.827	5.947
Q8NFH8	REPS2	9.437	15.999	9.841
P62993	GRB2	9.526	8.780	8.831
P31749	AKT	9.871	10.202	10.004
Q07889	SOS1	10.257	7.788	9.171
P51452	M3/6	10.807	10.265	12.322
P00519	ABL1	11.331	10.207	9.253
P05067	APP	11.646	13.139	9.236
P49768	PSEN1	11.843	7.844	6.508
O43639	NCK2	12.192	11.616	11.386
P60953	CDC42	13.341	12.561	11.683
P08913	ALPHA2AR	13.787	9.703	6.168
Q96RT1	ERBIN	14.186	9.313	11.085
P29353	SHC	14.732	13.282	17.058
Q96B97	SH3KBP1	17.578	12.601	12.491
Q9UNH7	SNX6	19.229	17.281	15.822
P46940	IQGAP	20.322	19.421	18.661
Q13882	PTK6	23.078	18.337	17.481
P18085	ARF4	36.423	32.452	34.981
P13688	CEACAM1	123.119	41.697	37.657

VEGFR signaling

UniProt	Name	Normal abundance	Adenoma abundance	Carcinoma abundance
Q15139	PKD	2.663	2.735	3.328
P06241	FYN	2.905	2.368	3.329
P35916	PCLy	3.221	3.231	3.486
P05106	ITGB3	3.320	3.468	3.644
P05771	nPKC	3.324	2.313	2.519
Q15759	MAPK11	3.605	3.460	3.340
Q9UQB8	IRSP53	3.792	4.370	4.134
Q05397	FAK	3.965	4.972	5.921
O75340	Alg-2	3.980	3.669	4.017
Q9ULV1	FZD4	4.122	3.779	4.537
Q14185	DOCK180	4.157	3.946	3.965
O14964	HGS	4.358	4.371	4.951
P18031	PTP1B	4.372	4.393	4.597
P35968	VEGFR	4.523	4.332	7.720
P04004	VITRONECTIN	4.650	4.594	4.353
Q13322	GRB10	4.914	6.006	6.803
P01584	IL1B	5.035	10.157	28.320
P42336	PI3K	5.087	4.688	5.842
P12931	SRC	5.240	5.415	5.119
P27986	P13K	5.390	5.584	4.955
P52735	VAV2	5.523	4.763	5.764

P56945	P130Cas	5.798	5.010	4.973
P15498	VAV	6.185	5.589	5.920
P49137	MAPKAP2	6.639	6.575	7.025
Q15464	EBS	6.662	10.194	10.280
Q14289	PTK	7.421	6.059	6.103
P15692	VEGF	7.524	8.043	12.599
P27540	ARNT	7.571	7.383	6.522
P07900	Hsp90	8.207	10.908	12.175
Q13464	ROCK1	8.858	9.267	8.337
O00459	p85beta	9.284	10.143	9.568
P49023	PXN	9.632	8.813	7.608
P19878	p67phox	9.899	8.391	20.058
Q9Y6W5	WAVE2	9.952	9.746	8.166
P42338	p110Ia	10.715	8.843	9.766
P04792	HSP27	10.930	8.181	10.793
P16333	NCK	11.062	10.723	10.211
Q16644	MAPKAP-K3	11.250	14.312	14.135
O43639	NCK2	12.192	11.616	11.386
P46108	CRK	12.906	11.606	10.953
O75116	ROCK2	13.225	14.308	12.839
P60953	CDC42	13.341	12.561	11.683
Q16539	p38	14.264	15.622	18.196
Q13177	PAK2	14.496	18.266	17.279
Q8IZP0	E3B1	15.342	17.323	17.234
Q16665	HIF1A	18.121	18.524	27.479
P63000	RAC1	19.531	21.914	23.407
P61586	RHOA	23.631	23.408	24.791
Q9UKW4	VAV3	66.036	54.527	52.007

mismatch repair

UniProt	Name	Normal abundance	Adenoma abundance	Carcinoma abundance
P05129	PRKCG	4.289	4.360	4.045
O15350	P73	4.298	4.486	4.097
Q9Y2T1	AXIN2	5.108	13.637	6.641
Q9NSU2	ATRIP	8.251	8.754	8.721
Q15054	p68	8.815	9.654	9.494
P00519	ABL1	11.331	10.207	9.253
P09429	HMG1	14.850	19.147	20.809
P12004	PCNA	37.079	62.586	71.442

Table S10. Median weighted degrees of targeted and immunotherapy pathway related nodes

Table S10. Median relative weighted degrees of targeted and immunotherapy pathway related nodes

	[a]**Normal -** weighted degree	[b]**Normal -** relative weighted degree	[a]**Adenoma -** weighted degree	[b]**Adenoma -** relative weighted degree	[a]**Carcinoma -** weighted degree	[b]**Carcinoma -** relative weighted degree
**EGFR signaling**	14.229	*2.592*	14.080	*2.545*	14.207	*2.548*
**VEGFR signaling**	15.884	*2.894*	15.580	*2.817*	15.651	*2.807*
**mismatch repair**	5.840	*1.064*	6.063	*1.096*	5.807	*1.042*
**the whole network**	5.488	*1*	5.532	*1*	5.575	*1*

[a]Weighted degrees are calculated according to the traditional formula, such as the sum of the link weights belonging to each node.

[b]Relative weighted degrees are the quotients of the weighted degrees of each of the signaling pathways and the whole network.

Supporting Codes

Code S1. Calculating diameter for undirected network

```
import networkx as nx
import pdb
import math

# Specify Project Folder
folder = "D:/Bori/diameter/"

filepath = folder + "carcinoma.csv"

#Load all the networks as undirected networks

G = nx.read_weighted_edgelist(path = filepath, delimiter = ';')

#Load all the networks as directed networks

#Gu = nx.read_weighted_edgelist(path = filepath, delimiter = ';')
#G = nx.read_weighted_edgelist(path = filepath, delimiter =
';',create_using=nx.DiGraph())




#These are not complete networks, so this is the removal of the smaller,
isolated parts


"""
#Directed case

Gc = max(nx.connected_components(Gu), key=len)

for component in list(nx.connected_components(Gu)):
    if len(component)<len(Gc):
        for node in component:
            G.remove_node(node)
"""



#Undirected case

Gc = max(nx.connected_components(G), key=len)

for component in list(nx.connected_components(G)):
    if len(component)<len(Gc):
        for node in component:
            G.remove_node(node)
```

```
#Add reciprocal edge value attributes

for u,v,a in G.edges(data=True):
    G[u][v]['reciprocal_weight'] = 1 / G[u][v]['weight']

#Add logarithmic edge weights of normal weights

for u,v,a in G.edges(data=True):
    G[u][v]['logarithmic_weight'] = math.log(G[u][v]['weight'], 10)

#Add inverted edge value attributes

# 1.: find largest edge weight
max = sorted(G.edges(data=True),key= lambda x:
x[2]['weight'],reverse=True)[0][2]['weight']

# 2.: add the inverted weight as an attribute
for u,v,a in G.edges(data=True):
    G[u][v]['inverted_weight'] = max - G[u][v]['weight']

# Do a negative logarithmic mapping of the edge weights

# 1.: Calculate sum of weights for normalizing
# weightSum = G.size('weight')

# 2.: Add the negative logarithmic map weight as an attribute
for u,v,a in G.edges(data=True):
    G[u][v]['normalized_weight'] = G[u][v]['weight'] / max
    G[u][v]['negative_logarithmic_map_weight'] = math.log((G[u][v]['weight'] /
max), 10) * (-1)




    #Shortest path calculation

#With NetworkX shortest path function

path_GNX = nx.shortest_path(G, weight = 'negative_logarithmic_map_weight')
#'weight' for smallest edgeweight diameter; 'reciprocal_weight' for biggest
#path_GNX = nx.shortest_path(G)

#With NetworkX Dijkstra function

path_GD = dict(nx.all_pairs_dijkstra_path(G, weight =
'negative_logarithmic_map_weight')) #'weight' for smallest edgeweight diameter;
'reciprocal_weight' for biggest


#With NetworkX Johnson function

path_GJ = nx.johnson(G, weight = 'negative_logarithmic_map_weight')
#'weight' for smallest edgeweight diameter; 'reciprocal_weight' for biggest
```

```
#Diameters based on just the edge weights

#With NetworkX integrated function

print("\n")
print("Diameters based on just the edge weights:")
print("\n")
print("NetworkX integrated function")
print("\n")

wnx = 0
wnx_greatest = 0
wnxlg = 0
wnxlg_greatest = 0
negativeLogMapWeight = 0
negativeLogMapWeightMax = 0
nodelist = []
shortestPathLengths = []
shortestPathLengthsLg = []
shortestPathLengthsNegativeLogMap = []

for node1, node2 in path_GNX.items():
    for key in node2:
            #print(node2[key])
            for i in range(0, len(node2[key])-1):
                #print(node2[key][i], node2[key][i+1])
                wnx += G[node2[key][i]][node2[key][i+1]]["weight"]
                wnxlg += G[node2[key][i]][node2[key][i+1]]["logarithmic_weight"]
                negativeLogMapWeight += 
G[node2[key][i]][node2[key][i+1]]["negative_logarithmic_map_weight"]
            if negativeLogMapWeight > negativeLogMapWeightMax:
                nodelist = node2[key]
                wnx_greatest = wnx
                wnxlg_greatest = wnxlg
                negativeLogMapWeightMax = negativeLogMapWeight
#            print("Diameter: ", wnx)
            shortestPathLengths.append(wnx)
            wnx = 0
#            print("Diameter (logarithmic): ", wnxlg)
            shortestPathLengthsLg.append(wnxlg)
            wnxlg = 0
#            print("\n")
            shortestPathLengthsNegativeLogMap.append(negativeLogMapWeight)
            negativeLogMapWeight = 0


print(nodelist)
print("\n")
print("Diameter: ", wnx_greatest)
print("Diameter (logarithmic): ", wnxlg_greatest)
print("Diameter (negative logarithmic map): ", negativeLogMapWeightMax)
print("Diameter (edge count): ", len(nodelist)-1)
print("Average shortest path length: ", sum(shortestPathLengths) / 
len(shortestPathLengths))
```

```
print("Average shortest path length (logarithmic): ", sum(shortestPathLengthsLg)
/ len(shortestPathLengthsLg))
print("Average shortest path length (negative logarithmic map): ",
sum(shortestPathLengthsNegativeLogMap) / len(shortestPathLengthsNegativeLogMap))


wnx = 0
wnxlg = 0


#With NetworkX Dijkstra function

print("\n")
print("NetworkX Dijkstra function")
print("\n")

wnx = 0
wnx_greatest = 0
wnxlg = 0
wnxlg_greatest = 0
negativeLogMapWeight = 0
negativeLogMapWeightMax = 0
nodelist = []
shortestPathLengths = []
shortestPathLengthsLg = []
shortestPathLengthsNegativeLogMap = []

for node1D, node2D in path_GD.items():
    for keyD in node2D:
            for i in range(0, len(node2D[keyD])-1):
                wnx += G[node2D[keyD][i]][node2D[keyD][i+1]]["weight"]
                wnxlg +=
G[node2D[keyD][i]][node2D[keyD][i+1]]["logarithmic_weight"]
                negativeLogMapWeight +=
G[node2D[keyD][i]][node2D[keyD][i+1]]["negative_logarithmic_map_weight"]
            if negativeLogMapWeight > negativeLogMapWeightMax:
                nodelist = node2D[keyD]
                wnx_greatest = wnx
                wnxlg_greatest = wnxlg
                negativeLogMapWeightMax = negativeLogMapWeight
            shortestPathLengths.append(wnx)
            wnx = 0
            shortestPathLengthsLg.append(wnxlg)
            wnxlg = 0
            shortestPathLengthsNegativeLogMap.append(negativeLogMapWeight)
            negativeLogMapWeight = 0

print(nodelist)
print("\n")
print("Diameter: ", wnx_greatest)
print("Diameter (logarithmic): ", wnxlg_greatest)
print("Diameter (negative logarithmic map): ", negativeLogMapWeightMax)
print("Diameter (edge count): ", len(nodelist)-1)
print("Average shortest path length: ", sum(shortestPathLengths) /
len(shortestPathLengths))
print("Average shortest path length (logarithmic): ", sum(shortestPathLengthsLg)
/ len(shortestPathLengthsLg))
print("Average shortest path length (negative logarithmic map): ",
sum(shortestPathLengthsNegativeLogMap) / len(shortestPathLengthsNegativeLogMap))
```

```
#With NetworkX Johnson function

print("\n")
print("NetworkX Johnson function")
print("\n")

wnx = 0
wnx_greatest = 0
wnxlg = 0
wnxlg_greatest = 0
negativeLogMapWeight = 0
negativeLogMapWeightMax = 0
nodelist = []
shortestPathLengths = []
shortestPathLengthsLg = []
shortestPathLengthsNegativeLogMap = []

for node1J, node2J in path_GJ.items():
    for keyJ in node2J:
            for i in range(0, len(node2J[keyJ])-1):
                wnx += G[node2J[keyJ][i]][node2J[keyJ][i+1]]["weight"]
                wnxlg +=
G[node2J[keyJ][i]][node2J[keyJ][i+1]]["logarithmic_weight"]
                negativeLogMapWeight +=
G[node2J[keyJ][i]][node2J[keyJ][i+1]]["negative_logarithmic_map_weight"]
            if negativeLogMapWeight > negativeLogMapWeightMax:
                nodelist = node2J[keyJ]
                wnx_greatest = wnx
                wnxlg_greatest = wnxlg
                negativeLogMapWeightMax = negativeLogMapWeight
            shortestPathLengths.append(wnx)
            wnx = 0
            shortestPathLengthsLg.append(wnxlg)
            wnxlg = 0
            shortestPathLengthsNegativeLogMap.append(negativeLogMapWeight)
            negativeLogMapWeight = 0

print(nodelist)
print("\n")
print("Diameter: ", wnx_greatest)
print("Diameter (logarithmic): ", wnxlg_greatest)
print("Diameter (negative logarithmic map): ", negativeLogMapWeightMax)
print("Diameter (edge count): ", len(nodelist)-1)
print("Average shortest path length: ", sum(shortestPathLengths) /
len(shortestPathLengths))
print("Average shortest path length (logarithmic): ", sum(shortestPathLengthsLg)
/ len(shortestPathLengthsLg))
print("Average shortest path length (negative logarithmic map): ",
sum(shortestPathLengthsNegativeLogMap) / len(shortestPathLengthsNegativeLogMap))
```

Code S2. Calculating diameter for directed network

```
import networkx as nx
import pdb
import math

# Specify Project Folder
folder = "D:/Bori/diameter/"

filepath = folder + "carcinoma.csv"

#Load all the networks as undirected networks

#G = nx.read_weighted_edgelist(path = filepath, delimiter = ';')

#Load all the networks as directed networks

Gu = nx.read_weighted_edgelist(path = filepath, delimiter = ';')
G = nx.read_weighted_edgelist(path = filepath, delimiter =
';',create_using=nx.DiGraph())




#These are not complete networks, so this is the removal of the smaller,
isolated parts


#Directed case

Gc = max(nx.connected_components(Gu), key=len)

for component in list(nx.connected_components(Gu)):
    if len(component)<len(Gc):
        for node in component:
            G.remove_node(node)



"""
#Undirected case

Gc = max(nx.connected_components(G), key=len)

for component in list(nx.connected_components(G)):
    if len(component)<len(Gc):
        for node in component:
            G.remove_node(node)
"""
```

```
#Add reciprocal edge value attributes

for u,v,a in G.edges(data=True):
    G[u][v]['reciprocal_weight'] = 1 / G[u][v]['weight']

#Add logarithmic edge weights of normal weights

for u,v,a in G.edges(data=True):
    G[u][v]['logarithmic_weight'] = math.log(G[u][v]['weight'], 10)

#Add inverted edge value attributes

# 1.: find largest edge weight
max = sorted(G.edges(data=True),key= lambda x:
x[2]['weight'],reverse=True)[0][2]['weight']

# 2.: add the inverted weight as an attribute
for u,v,a in G.edges(data=True):
    G[u][v]['inverted_weight'] = max - G[u][v]['weight']

# Do a negative logarithmic mapping of the edge weights

# 1.: Calculate sum of weights for normalizing
# weightSum = G.size('weight')

# 2.: Add the negative logarithmic map weight as an attribute
for u,v,a in G.edges(data=True):
    G[u][v]['normalized_weight'] = G[u][v]['weight'] / max
    G[u][v]['negative_logarithmic_map_weight'] = math.log((G[u][v]['weight'] /
max), 10) * (-1)




    #Shortest path calculation

#With NetworkX shortest path function

path_GNX = nx.shortest_path(G, weight = 'negative_logarithmic_map_weight')
#'weight' for smallest edgeweight diameter; 'reciprocal_weight' for biggest
#path_GNX = nx.shortest_path(G)

#With NetworkX Dijkstra function

path_GD = dict(nx.all_pairs_dijkstra_path(G, weight =
'negative_logarithmic_map_weight')) #'weight' for smallest edgeweight diameter;
'reciprocal_weight' for biggest


#With NetworkX Johnson function

path_GJ = nx.johnson(G, weight = 'negative_logarithmic_map_weight')
#'weight' for smallest edgeweight diameter; 'reciprocal_weight' for biggest
```

```
#Diameters based on just the edge weights

#With NetworkX integrated function

print("\n")
print("Diameters based on just the edge weights:")
print("\n")
print("NetworkX integrated function")
print("\n")

wnx = 0
wnx_greatest = 0
wnxlg = 0
wnxlg_greatest = 0
negativeLogMapWeight = 0
negativeLogMapWeightMax = 0
nodelist = []
shortestPathLengths = []
shortestPathLengthsLg = []
shortestPathLengthsNegativeLogMap = []

for node1, node2 in path_GNX.items():
    for key in node2:
            #print(node2[key])
            for i in range(0, len(node2[key])-1):
                #print(node2[key][i], node2[key][i+1])
                wnx += G[node2[key][i]][node2[key][i+1]]["weight"]
                wnxlg += G[node2[key][i]][node2[key][i+1]]["logarithmic_weight"]
                negativeLogMapWeight +=
G[node2[key][i]][node2[key][i+1]]["negative_logarithmic_map_weight"]
            if negativeLogMapWeight > negativeLogMapWeightMax:
                nodelist = node2[key]
                wnx_greatest = wnx
                wnxlg_greatest = wnxlg
                negativeLogMapWeightMax = negativeLogMapWeight
#            print("Diameter: ", wnx)
            shortestPathLengths.append(wnx)
            wnx = 0
#            print("Diameter (logarithmic): ", wnxlg)
            shortestPathLengthsLg.append(wnxlg)
            wnxlg = 0
#            print("\n")
            shortestPathLengthsNegativeLogMap.append(negativeLogMapWeight)
            negativeLogMapWeight = 0


print(nodelist)
print("\n")
print("Diameter: ", wnx_greatest)
print("Diameter (logarithmic): ", wnxlg_greatest)
print("Diameter (negative logarithmic map): ", negativeLogMapWeightMax)
print("Diameter (edge count): ", len(nodelist)-1)
print("Average shortest path length: ", sum(shortestPathLengths) /
len(shortestPathLengths))
print("Average shortest path length (logarithmic): ", sum(shortestPathLengthsLg)
/ len(shortestPathLengthsLg))
print("Average shortest path length (negative logarithmic map): ",
sum(shortestPathLengthsNegativeLogMap) / len(shortestPathLengthsNegativeLogMap))
```

```
wnx = 0
wnxlg = 0


#With NetworkX Dijkstra function

print("\n")
print("NetworkX Dijkstra function")
print("\n")

wnx = 0
wnx_greatest = 0
wnxlg = 0
wnxlg_greatest = 0
negativeLogMapWeight = 0
negativeLogMapWeightMax = 0
nodelist = []
shortestPathLengths = []
shortestPathLengthsLg = []
shortestPathLengthsNegativeLogMap = []

for node1D, node2D in path_GD.items():
    for keyD in node2D:
            for i in range(0, len(node2D[keyD])-1):
                wnx += G[node2D[keyD][i]][node2D[keyD][i+1]]["weight"]
                wnxlg +=
G[node2D[keyD][i]][node2D[keyD][i+1]]["logarithmic_weight"]
                negativeLogMapWeight +=
G[node2D[keyD][i]][node2D[keyD][i+1]]["negative_logarithmic_map_weight"]
            if negativeLogMapWeight > negativeLogMapWeightMax:
                nodelist = node2D[keyD]
                wnx_greatest = wnx
                wnxlg_greatest = wnxlg
                negativeLogMapWeightMax = negativeLogMapWeight
            shortestPathLengths.append(wnx)
            wnx = 0
            shortestPathLengthsLg.append(wnxlg)
            wnxlg = 0
            shortestPathLengthsNegativeLogMap.append(negativeLogMapWeight)
            negativeLogMapWeight = 0

print(nodelist)
print("\n")
print("Diameter: ", wnx_greatest)
print("Diameter (logarithmic): ", wnxlg_greatest)
print("Diameter (negative logarithmic map): ", negativeLogMapWeightMax)
print("Diameter (edge count): ", len(nodelist)-1)
print("Average shortest path length: ", sum(shortestPathLengths) /
len(shortestPathLengths))
print("Average shortest path length (logarithmic): ", sum(shortestPathLengthsLg)
/ len(shortestPathLengthsLg))
print("Average shortest path length (negative logarithmic map): ",
sum(shortestPathLengthsNegativeLogMap) / len(shortestPathLengthsNegativeLogMap))



#With NetworkX Johnson function

print("\n")
print("NetworkX Johnson function")
```

```
print("\n")

wnx = 0
wnx_greatest = 0
wnxlg = 0
wnxlg_greatest = 0
negativeLogMapWeight = 0
negativeLogMapWeightMax = 0
nodelist = []
shortestPathLengths = []
shortestPathLengthsLg = []
shortestPathLengthsNegativeLogMap = []

for node1J, node2J in path_GJ.items():
    for keyJ in node2J:
            for i in range(0, len(node2J[keyJ])-1):
                wnx += G[node2J[keyJ][i]][node2J[keyJ][i+1]]["weight"]
                wnxlg += 
G[node2J[keyJ][i]][node2J[keyJ][i+1]]["logarithmic_weight"]
                negativeLogMapWeight += 
G[node2J[keyJ][i]][node2J[keyJ][i+1]]["negative_logarithmic_map_weight"]
            if negativeLogMapWeight > negativeLogMapWeightMax:
                nodelist = node2J[keyJ]
                wnx_greatest = wnx
                wnxlg_greatest = wnxlg
                negativeLogMapWeightMax = negativeLogMapWeight
            shortestPathLengths.append(wnx)
            wnx = 0
            shortestPathLengthsLg.append(wnxlg)
            wnxlg = 0
            shortestPathLengthsNegativeLogMap.append(negativeLogMapWeight)
            negativeLogMapWeight = 0

print(nodelist)
print("\n")
print("Diameter: ", wnx_greatest)
print("Diameter (logarithmic): ", wnxlg_greatest)
print("Diameter (negative logarithmic map): ", negativeLogMapWeightMax)
print("Diameter (edge count): ", len(nodelist)-1)
print("Average shortest path length: ", sum(shortestPathLengths) / 
len(shortestPathLengths))
print("Average shortest path length (logarithmic): ", sum(shortestPathLengthsLg) 
/ len(shortestPathLengthsLg))
print("Average shortest path length (negative logarithmic map): ", 
sum(shortestPathLengthsNegativeLogMap) / len(shortestPathLengthsNegativeLogMap))
```

Code S3. Calculating diameter for mixed network

```
import networkx as nx
import pandas as pd
import math


def read_create_MultiDiGraph(filename):

    G = nx.MultiDiGraph()

    data = pd.read_csv(filename, delimiter = ';')

    for index, row in data.iterrows():
        if row['Link_Type'] is "0":
            G.add_edge(row['From (Gene name)'], row['To (Gene name)'], weight =
row['Link_Weight'])
            G.add_edge(row['To (Gene name)'], row['From (Gene name)'], weight =
row['Link_Weight'])
        else:
            G.add_edge(row['From (Gene name)'], row['To (Gene name)'], weight =
row['Link_Weight'])
    return G


def remove_isolated_components(g, filename_u):
    G = g

    Gu = nx.read_weighted_edgelist(path = filename_u, delimiter = ';')
    Gc = max(nx.connected_components(Gu), key=len)

    for component in list(nx.connected_components(Gu)):
        if len(component)<len(Gc):
            for node in component:
                G.remove_node(node)
    return G

#Calculate diameter

def diameter(filename, filename_u):

    G = read_create_MultiDiGraph(filename)
    G = remove_isolated_components(G, filename_u)


    #Add reciprocal edge value attributes

    for u,v,a in G.edges(data=True):
        for key in G[u][v].keys():
            G[u][v][key]['reciprocal_weight'] = 1 / G[u][v][key]['weight']

    #Add logarithmic edge weights of normal weights

    for u,v,a in G.edges(data=True):
        for key in G[u][v].keys():
            G[u][v][key]['logarithmic_weight'] =
math.log(G[u][v][key]['weight'], 10)
```

```
    #Add inverted edge value attributes

    # 1.: find largest edge weight
    max = sorted(G.edges(data=True),key= lambda x:
x[2]['weight'],reverse=True)[0][2]['weight']

    # 2.: add the inverted weight as an attribute
    for u,v,a in G.edges(data=True):
        for key in G[u][v].keys():
            G[u][v][key]['inverted_weight'] = max - G[u][v][key]['weight']

    # Do a negative logarithmic mapping of the edge weights
    for u,v,a in G.edges(data=True):
        for key in G[u][v].keys():
            G[u][v][key]['normalized_weight'] = G[u][v][key]['weight'] / max
            G[u][v][key]['negative_logarithmic_map_weight'] =
math.log((G[u][v][key]['weight'] / max), 10) * (-1)



        #Shortest path calculation

    #With NetworkX shortest path function
    path_GNX = nx.shortest_path(G, weight = 'negative_logarithmic_map_weight' )

    #With NetworkX Dijkstra function
    path_GD = dict(nx.all_pairs_dijkstra_path(G, weight =
'negative_logarithmic_map_weight' ))


    #With NetworkX Johnson function
    path_GJ = nx.johnson(G, weight = 'negative_logarithmic_map_weight' )


    #Diameters based on just the edge weights

    #With NetworkX integrated function

    print("\n")
    print("Diameters based on just the edge weights:")
    print("\n")
    print("NetworkX integrated function")
    print("\n")

    wnx = 0
    wnx_greatest = 0
    wnxlg = 0
    wnxlg_greatest = 0
    negativeLogMapWeight = 0
    negativeLogMapWeightMax = 0
    nodelist = []
    shortestPathLengths = []
    shortestPathLengthsLg = []
    shortestPathLengthsNegativeLogMap = []

    for node1, node2 in path_GNX.items():
        for key in node2:
                for i in range(0, len(node2[key])-1):
                    wnx += G[node2[key][i]][node2[key][i+1]][0]["weight"]
                    wnxlg +=
G[node2[key][i]][node2[key][i+1]][0]["logarithmic_weight"]
```

```
                        negativeLogMapWeight +=
G[node2[key][i]][node2[key][i+1]][0]["negative_logarithmic_map_weight"]
                    if negativeLogMapWeight > negativeLogMapWeightMax:
                        nodelist = node2[key]
                        wnx_greatest = wnx
                        wnxlg_greatest = wnxlg
                        negativeLogMapWeightMax = negativeLogMapWeight
    #                print("Diameter: ", wnx)
                    shortestPathLengths.append(wnx)
                    wnx = 0
    #                print("Diameter (logarithmic): ", wnxlg)
                    shortestPathLengthsLg.append(wnxlg)
                    wnxlg = 0
    #                print("\n")
                    shortestPathLengthsNegativeLogMap.append(negativeLogMapWeight)
                    negativeLogMapWeight = 0


    print(nodelist)
    print("\n")
    print("Diameter: ", wnx_greatest)
    print("Diameter (logarithmic): ", wnxlg_greatest)
    print("Diameter (negative logarithmic map): ", negativeLogMapWeightMax)
    print("Diameter (edge count): ", len(nodelist)-1)
    print("Average shortest path length: ", sum(shortestPathLengths) /
len(shortestPathLengths))
    print("Average shortest path length (logarithmic): ",
sum(shortestPathLengthsLg) / len(shortestPathLengthsLg))
    print("Average shortest path length (negative logarithmic map): ",
sum(shortestPathLengthsNegativeLogMap) / len(shortestPathLengthsNegativeLogMap))

    wnx = 0
    wnxlg = 0


    #With NetworkX Dijkstra function

    print("\n")
    print("NetworkX Dijkstra function")
    print("\n")

    wnx = 0
    wnx_greatest = 0
    wnxlg = 0
    wnxlg_greatest = 0
    negativeLogMapWeight = 0
    negativeLogMapWeightMax = 0
    nodelist = []
    shortestPathLengths = []
    shortestPathLengthsLg = []
    shortestPathLengthsNegativeLogMap = []

    for node1D, node2D in path_GD.items():
        for keyD in node2D:
                for i in range(0, len(node2D[keyD])-1):
                    wnx += G[node2D[keyD][i]][node2D[keyD][i+1]][0]["weight"]
                    wnxlg +=
G[node2D[keyD][i]][node2D[keyD][i+1]][0]["logarithmic_weight"]
                    negativeLogMapWeight +=
G[node2D[keyD][i]][node2D[keyD][i+1]][0]["negative_logarithmic_map_weight"]
                if negativeLogMapWeight > negativeLogMapWeightMax:
```

```
                    nodelist = node2D[keyD]
                    wnx_greatest = wnx
                    wnxlg_greatest = wnxlg
                    negativeLogMapWeightMax = negativeLogMapWeight
                shortestPathLengths.append(wnx)
                wnx = 0
                shortestPathLengthsLg.append(wnxlg)
                wnxlg = 0
                shortestPathLengthsNegativeLogMap.append(negativeLogMapWeight)
                negativeLogMapWeight = 0

    print(nodelist)
    print("\n")
    print("Diameter: ", wnx_greatest)
    print("Diameter (logarithmic): ", wnxlg_greatest)
    print("Diameter (negative logarithmic map): ", negativeLogMapWeightMax)
    print("Diameter (edge count): ", len(nodelist)-1)
    print("Average shortest path length: ", sum(shortestPathLengths) /
len(shortestPathLengths))
    print("Average shortest path length (logarithmic): ",
sum(shortestPathLengthsLg) / len(shortestPathLengthsLg))
    print("Average shortest path length (negative logarithmic map): ",
sum(shortestPathLengthsNegativeLogMap) / len(shortestPathLengthsNegativeLogMap))



    #With NetworkX Johnson function

    print("\n")
    print("NetworkX Johnson function")
    print("\n")

    wnx = 0
    wnx_greatest = 0
    wnxlg = 0
    wnxlg_greatest = 0
    negativeLogMapWeight = 0
    negativeLogMapWeightMax = 0
    nodelist = []
    shortestPathLengths = []
    shortestPathLengthsLg = []
    shortestPathLengthsNegativeLogMap = []

    for node1J, node2J in path_GJ.items():
        for keyJ in node2J:
                for i in range(0, len(node2J[keyJ])-1):
                    wnx += G[node2J[keyJ][i]][node2J[keyJ][i+1]][0]["weight"]
                    wnxlg +=
G[node2J[keyJ][i]][node2J[keyJ][i+1]][0]["logarithmic_weight"]
                    negativeLogMapWeight +=
G[node2J[keyJ][i]][node2J[keyJ][i+1]][0]["negative_logarithmic_map_weight"]
                if negativeLogMapWeight > negativeLogMapWeightMax:
                    nodelist = node2J[keyJ]
                    wnx_greatest = wnx
                    wnxlg_greatest = wnxlg
                    negativeLogMapWeightMax = negativeLogMapWeight
                shortestPathLengths.append(wnx)
                wnx = 0
                shortestPathLengthsLg.append(wnxlg)
                wnxlg = 0
                shortestPathLengthsNegativeLogMap.append(negativeLogMapWeight)
```

```
                negativeLogMapWeight = 0

    print(nodelist)
    print("\n")
    print("Diameter: ", wnx_greatest)
    print("Diameter (logarithmic): ", wnxlg_greatest)
    print("Diameter (negative logarithmic map): ", negativeLogMapWeightMax)
    print("Diameter (edge count): ", len(nodelist)-1)
    print("Average shortest path length: ", sum(shortestPathLengths) /
len(shortestPathLengths))
    print("Average shortest path length (logarithmic): ",
sum(shortestPathLengthsLg) / len(shortestPathLengthsLg))
    print("Average shortest path length (negative logarithmic map): ",
sum(shortestPathLengthsNegativeLogMap) / len(shortestPathLengthsNegativeLogMap))




diameter('carcinoma.csv', 'carcinoma_u.csv')
```
